# Genome-Wide Identification of the Maize TALE Gene Family and Their Expression Analysis Under Low-Phosphorus Response in Maize (*Zea mays* L.)

**DOI:** 10.3390/plants15162444

**Published:** 2026-08-11

**Authors:** Xianting Huang, Shuang Li, Litao Yi, Aiping Yin, Qingtao Zeng, Han Lv, Feiyan Li, Zengqiang Meng, Chaofeng Li, Xiupeng Mei, Jiuguang Wang

**Affiliations:** 1 Maize Research Institute, Southwest University, Chongqing 400715, China; 13062355750@163.com (X.H.); ls18855077417@126.com (S.L.); 13068332389@163.com (L.Y.); yaaap8910@163.com (A.Y.); 15893521629@163.com (Q.Z.); lv03162046@163.com (H.L.); lifeiyan200103@163.com (F.L.); 17782177173@163.com (Z.M.); cfli1988cas@swu.edu.cn (C.L.); 2 College of Agronomy and Biotechnology, Southwest University, Chongqing 400715, China; 3 Engineering Research Center of South Upland Agriculture, Ministry of Education, Chongqing 400715, China

**Keywords:** *Zea mays*, TALE transcription factor, bioinformatics, low-phosphorus response

## Abstract

The three-amino-acid-loop-extension (TALE) gene family encodes a group of plant-specific homeodomain transcription factors that play indispensable roles in plant growth, development, and adaptation to environmental stresses. Although TALE genes have been extensively investigated in several plant species, their genome-wide characteristics and potential functions in maize, particularly in response to phosphorus deficiency, remain poorly understood. In the present study, a comprehensive genome-wide identification and characterization of the maize TALE gene family were conducted using bioinformatics approaches, followed by an investigation of their transcriptional responses to low-phosphorus (LP) stress. A total of 40 *ZmTALE* genes (ZmTALE1–ZmTALE40) were identified and phylogenetically classified into four subfamilies: BEL1-like, KNOX I, KNOX II, and KNOX III. Members within the same subfamily exhibited highly conserved gene structures and motif compositions, reflecting their evolutionary conservation. Chromosomal localization and synteny analyses demonstrated that segmental duplication has been the predominant force driving the expansion of the ZmTALE gene family during maize evolution. Promoter analysis revealed that the upstream regulatory regions of *ZmTALE* genes were enriched in light-responsive, phytohormone-responsive, and abiotic stress-related cis-acting regulatory elements, implying their potential involvement in multiple developmental and stress-responsive pathways. Expression profiling under LP conditions revealed pronounced genotype-dependent transcriptional responses among different maize inbred lines. Notably, *ZmTALE1/5/12/14/18/30/31/33/36* were significantly induced by LP stress, whereas *ZmTALE10* and *ZmTALE37* were markedly repressed. These differentially expressed genes represent promising candidates for further functional investigation of phosphorus-deficiency tolerance in maize. Furthermore, ZmTALE10, ZmTALE14, and ZmTALE31 are nuclear-localized transcriptional activators. Taken together, these findings provide valuable insights into the evolutionary characteristics and potential biological functions of the maize TALE gene family and offer candidate genes for developing phosphorus-efficient maize cultivars through molecular breeding.

## 1. Introduction

The three-amino-acid-loop-extension (TALE) gene family encodes a class of plant-specific homeodomain transcription factors characterized by the insertion of three additional amino acids within the homeodomain [1]. Based on sequence features and evolutionary relationships, TALE proteins are classified into the KNOTTED1-like homeobox (KNOX) and BEL1-like homeobox (BELL) subfamilies [2], whose members frequently form homo- or heterodimeric complexes to regulate downstream gene expression. TALE transcription factors play essential roles in diverse developmental processes, including shoot apical meristem maintenance, organogenesis, embryogenesis, leaf morphogenesis, vascular development, and reproductive growth [3,4,5,6]. Recent studies have further demonstrated that TALE proteins contribute to plant adaptation to abiotic stresses, such as drought, salinity, cold, and nutrient deficiency, through the regulation of root development, hormone signaling, cell differentiation, and stress-responsive gene expression [7,8,9,10]. They also interact with multiple phytohormone pathways, including auxin, cytokinin, gibberellin, and abscisic acid, to coordinate plant growth with environmental adaptation [11,12]. Although the functions of *TALE* genes have been extensively investigated in model plants, their evolutionary characteristics and stress-related functions in maize remain poorly understood [13,14].

Phosphorus (P) is an essential macronutrient required for energy metabolism, nucleic acid synthesis, membrane biogenesis, and intracellular signaling [15,16]. However, phosphate availability is often limited because most inorganic phosphate in soils exists in insoluble forms [17], making phosphorus deficiency a major constraint on maize productivity worldwide. To cope with phosphate starvation, plants have evolved complex adaptive mechanisms, including remodeling root architecture, enhancing phosphate uptake and remobilization, and reprogramming gene expression [18,19,20]. Central regulators of these responses include the PHR–SPX signaling module, PHT1 phosphate transporters, and several transcription factor families such as WRKY, MYB, NAC, and bHLH [21,22,23]. By contrast, the involvement of TALE transcription factors in phosphorus-deficiency responses has received little attention, particularly in maize.

In this study, we conducted a comprehensive genome-wide analysis of the maize TALE gene family. Phylogenetic relationships, chromosomal distribution, gene structures, conserved motifs, gene duplication events, syntenic relationships, and promoter cis-regulatory elements were systematically analyzed. In addition, tissue-specific expression patterns and transcriptional responses to low-phosphorus stress were investigated to identify candidate genes potentially involved in phosphorus-deficiency adaptation. These results provide new insights into the evolution and potential biological functions of maize TALE genes and establish a foundation for future functional characterization and the molecular breeding of phosphorus-efficient maize cultivars.

## 2. Results

### 2.1. Identification and Characterization of the Maize TALE Gene Family

A total of 40 *TALE* genes were ultimately identified in the maize genome and designated ZmTALE1-ZmTALE40 according to their physical positions on the chromosomes (Table 1). The physicochemical properties of the predicted ZmTALE proteins are summarized in Table 1. The protein lengths varied substantially, ranging from 93 to 755 amino acids (aa), with predicted molecular weights (MWs) ranging from 10.58 to 79.17 kDa, indicating considerable structural diversity among family members. The theoretical isoelectric points (pIs) ranged from 4.59 to 9.24, suggesting that ZmTALE proteins possess diverse physicochemical characteristics. Among them, ZmTALE24 (pI = 4.59) and ZmTALE6 (pI = 4.88) were predicted to be acidic proteins, whereas ZmTALE33 (pI = 9.24) and ZmTALE39 (pI = 9.23) were predicted to be basic proteins. The grand average of hydropathicity (GRAVY) values ranged from −0.762 to −0.038, indicating that all ZmTALE proteins are hydrophilic. Analysis of the instability index revealed that, with the exception of a few proteins, including ZmTALE16 (38.40) and ZmTALE12 (39.64), most ZmTALE proteins had instability index values greater than 40, suggesting that they are predicted to be unstable proteins. Signal peptide prediction further showed that all ZmTALE proteins exhibited SignalP scores well below the threshold value of 0.5, indicating that they are likely non-secretory proteins.

Subcellular localization prediction indicated that 30 of the 40 ZmTALE proteins (75.0%) were localized to the nucleus, which is consistent with their putative roles as transcription factors (Table 1). Notably, ZmTALE4 was predicted to localize to the endoplasmic reticulum (ER), ZmTALE15, ZmTALE16, and ZmTALE34 to the chloroplast, and ZmTALE24, ZmTALE39, and ZmTALE40 to the mitochondrion. These distinct subcellular localization patterns suggest that certain ZmTALE proteins may possess specialized biological functions or participate in inter-organelle signaling and regulatory processes.

### 2.2. Chromosomal Distribution and Structural Characteristics of ZmTALE Proteins

**Figure 1:**
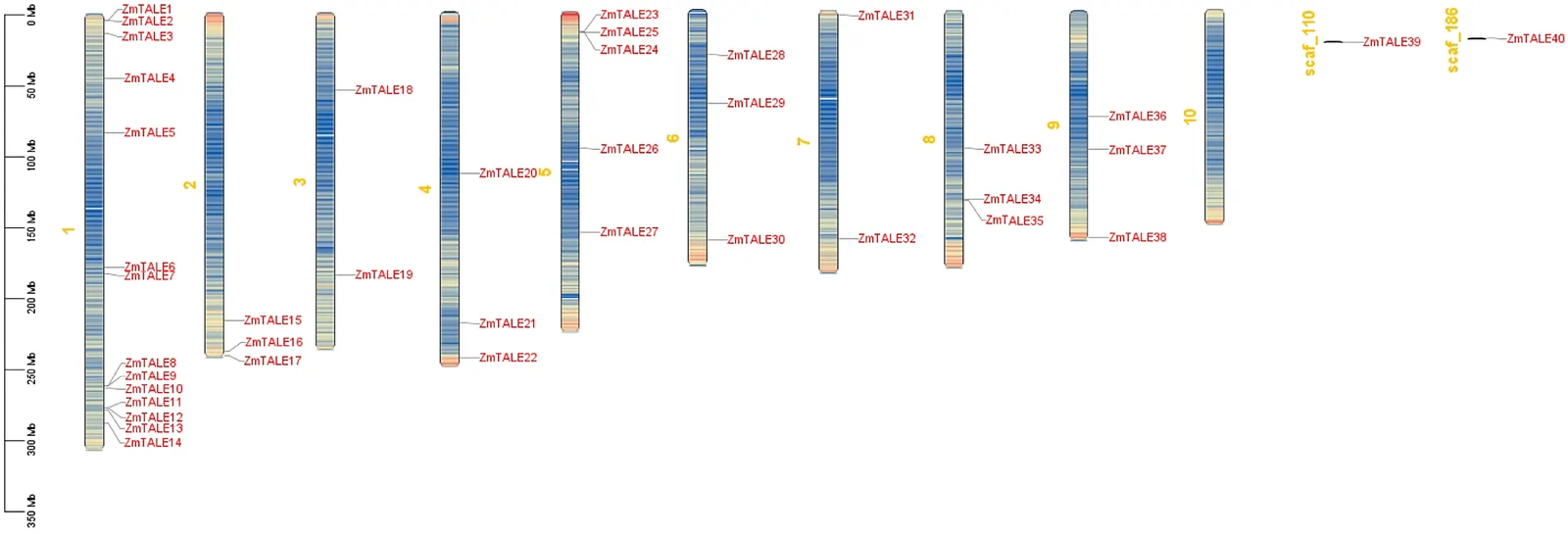
**Chromosome location of TALE gene family in *Zea Mays*.** The lines indicate chromosomal positions harboring TALE genes, and the gene names in red correspond to the respective TALE loci. The number to the left of the chromosome indicates the chromosome number. The different colors on the chromosome show the density of genes.

Chromosomal localization analysis revealed that the 40 *ZmTALE* genes were unevenly distributed across the maize genome (Figure 1). A total of 38 *ZmTALE* genes were mapped to nine chromosomes, whereas no *TALE* gene was identified on chromosome 10. The remaining two genes were located on unanchored scaffolds. Chromosome 1 contained the largest number of *ZmTALE* genes (14 members), followed by chromosome 5 with five members. Chromosomes 2, 4, 6, 8, and 9 each harbored three *ZmTALE* genes, whereas chromosomes 3 and 7 each contained two members (Figure 1), indicating an uneven chromosomal distribution of the ZmTALE gene family.

The results further revealed that members of the ZmTALE gene family exhibit clustered chromosomal distribution. For instance, ZmTALE1-ZmTALE5 are densely clustered within the terminal region of the short arm of chromosome 1 (Chr1), spanning the physical interval of 0–50 Mb; ZmTALE8–ZmTALE14 are concentrated at the distal end of the long arm of Chr1, corresponding to the 250–300 Mb genomic segment. In addition, ZmTALE23 and ZmTALE24 form a gene cluster at the tip of the short arm of chromosome 5 (Chr5) within the 0–50 Mb interval (Figure 1). Notably, two distinct gene-rich regions were identified on Chr1, implying that this chromosome has likely undergone frequent segmental duplication events, which drove the expansion and evolutionary diversification of the TALE gene family in the maize genome.

Secondary structure prediction showed that all 40 ZmTALE proteins contained α-helices (α-helices) and random coils, whereas β-turns were absent in all family members. With the exception of ZmTALE6, ZmTALE15, ZmTALE20, ZmTALE25, ZmTALE37, and ZmTALE39, all other ZmTALE proteins also contained extended β-strands. Among the predicted secondary structural elements, random coils represented the predominant component, accounting for approximately 80% of the total secondary structure content (Supplementary Table S1), suggesting that intrinsically disordered coil regions constitute the major structural framework of ZmTALE proteins.

**Figure 2:**
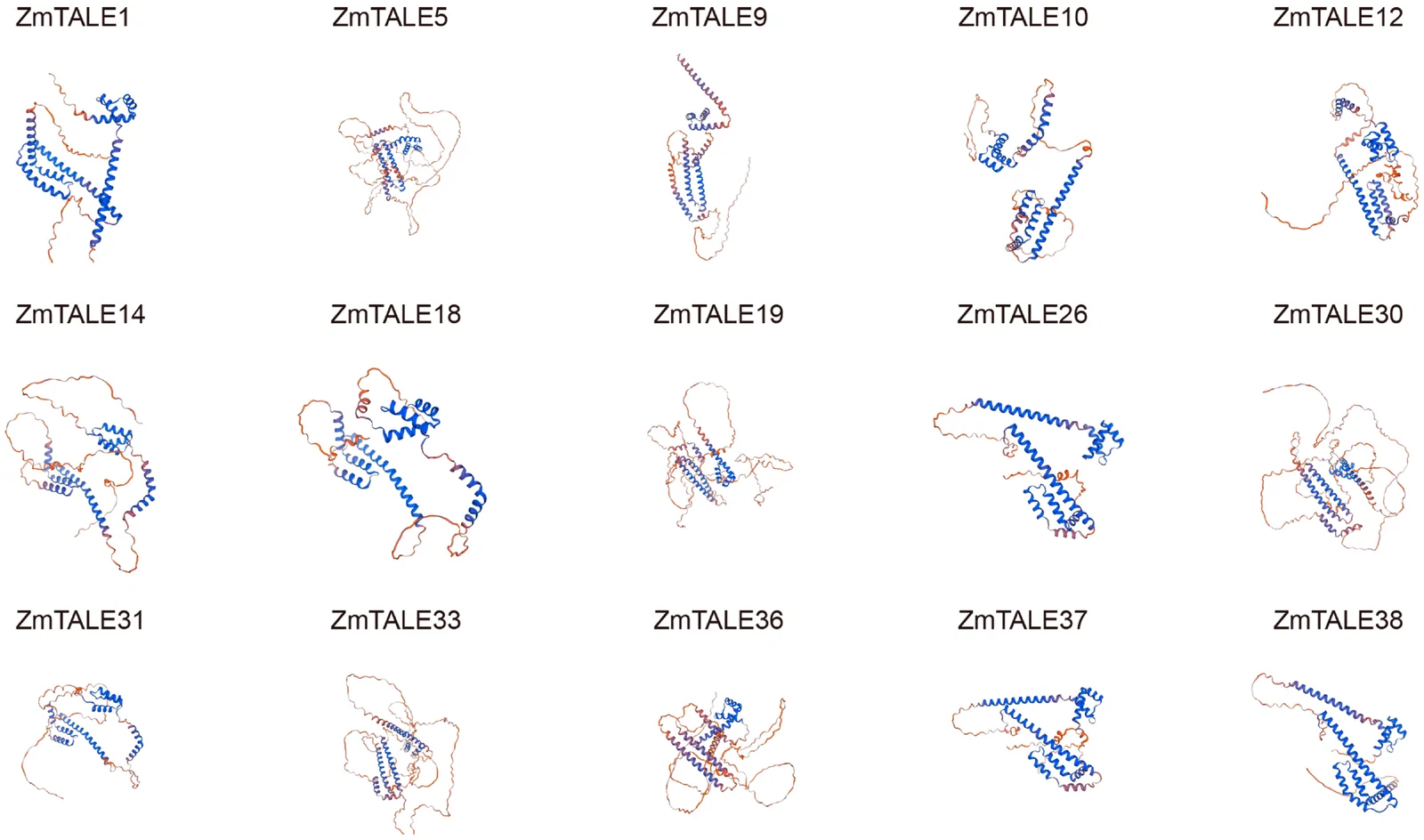
**Predicted 3D structures of selected maize TALE proteins.** Blue color represents the N-terminus of the amino acid sequence, and red color represents the C-terminus of the amino acid sequence. Then, the blue part is an alpha-helix.

To further characterize the structural features of ZmTALE proteins, three-dimensional (3D) structures were predicted by homology modeling. The predicted models demonstrated a high degree of structural similarity among proteins belonging to the same subfamily. For example, ZmTALE28 and ZmTALE35 (KNOX I), ZmTALE1 and ZmTALE38 (KNOX II), ZmTALE15, ZmTALE20, and ZmTALE24 (KNOX III), ZmTALE39 and ZmTALE40, as well as ZmTALE8 and ZmTALE23 (BEL1-like), exhibited highly similar three-dimensional conformations, with several protein pairs displaying nearly identical structural architectures (Figure 2 and Supplementary Figure S1). Except for ZmTALE6, ZmTALE39, and ZmTALE40, which lacked portions of the conserved KNOX domain, all remaining ZmTALE proteins exhibited highly conserved spatial arrangements of the KNOX domain. Overall, the predicted tertiary structures further confirmed that ZmTALE proteins are predominantly composed of α-helices, extended β-strands, and random coils, with no β-turns detected in any family member (Figure 2 and Supplementary Figure S1).

### 2.3. Phylogenetic Characterization and Classification for the ZmTALE Gene Family 

**Figure 3:**
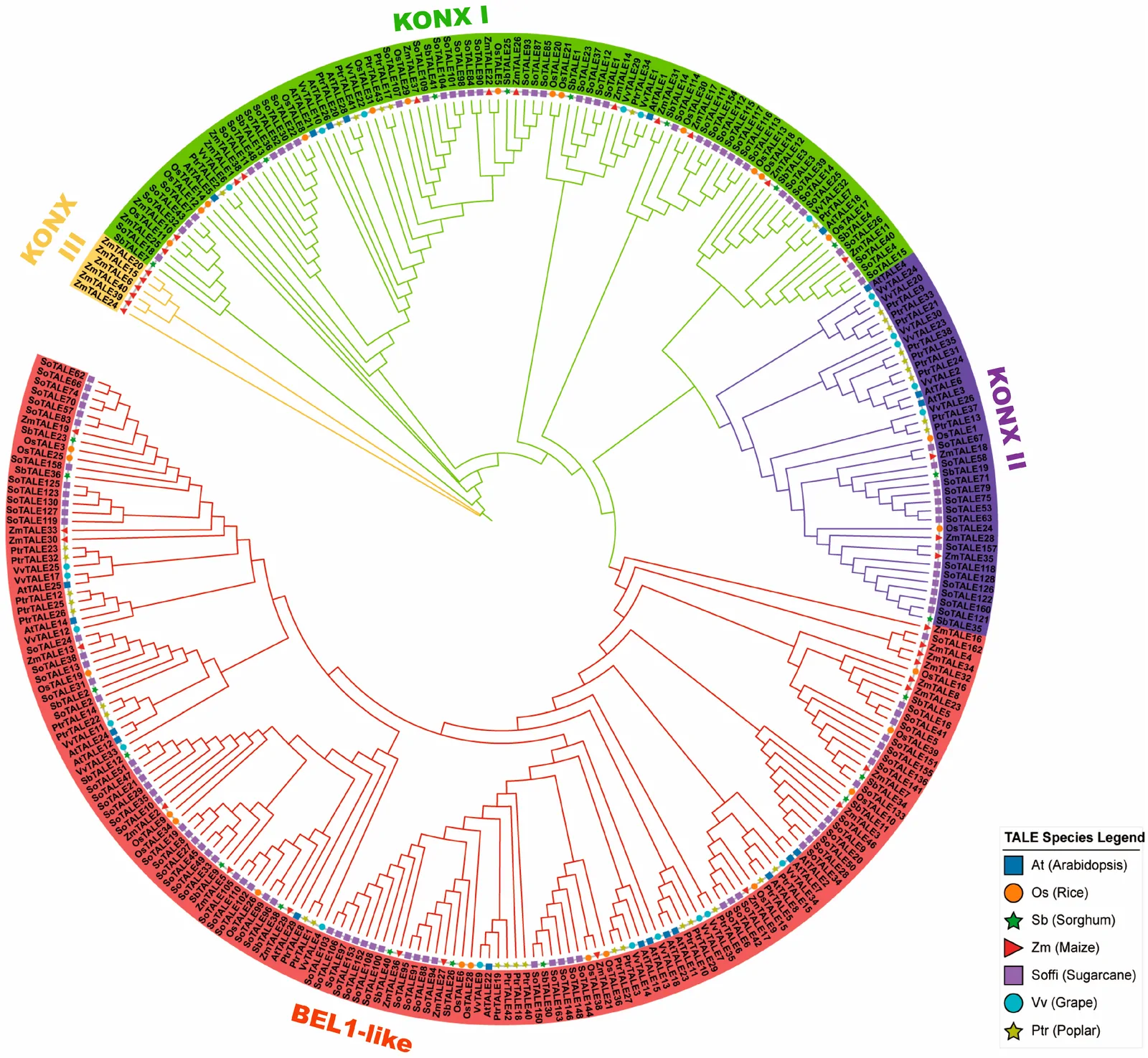
**Phylogenetic tree of the TALE gene family in seven species.** An unrooted phylogenetic tree was constructed using the maximum likelihood (ML) method with 1000 bootstrap replicates. Different shapes and colors represent different species: a red triangle represents maize (*Zea mays*), a blue square represents *Arabidopsis thaliana*, a green pentagram represents sorghum (*Sorghum bicolor*), an orange circle represents rice (*Oryza sativa*), a purple square represents sugarcane (*Saccharum officinarum*), a yellow-green pentagram represents poplar (*Populus trichocarpa*), and a light blue circle represents grape (*Vitis vinifera*).

To investigate the evolutionary relationships and classification of the ZmTALE gene family, TALE protein sequences from seven representative plant species, including maize (*Zea mays*), *A. thaliana*, rice (*O. sativa*), sorghum (*Sorghum bicolor*), sugarcane (*Saccharum officinarum*), poplar (*Populus trichocarpa*), and grapevine (*Vitis vinifera*), were collected for phylogenetic analysis. Multiple sequence alignment was performed using ClustalW, and an unrooted phylogenetic tree was constructed using the maximum likelihood (ML) method with 1000 bootstrap replicates (Figure 3). The ML tree showed clear clustering patterns and classified TALE proteins into four well-supported subfamilies: BEL1-like, KNOX I, KNOX II, and KNOX III, consistent with previous classifications reported in Arabidopsis and other plant species, indicating that the TALE family is evolutionarily conserved among diverse plant lineages.

The phylogenetic analysis clearly classified all TALE proteins into four well-supported subfamilies: BEL1-like, KNOX I, KNOX II, and KNOX III, which is consistent with the classification previously reported in *Arabidopsis*, *Medicago truncatula* [24], and other plant species, indicating that the TALE gene family is evolutionarily conserved across the plant kingdom. Based on the phylogenetic analysis, all 40 ZmTALE proteins were classified into four subfamilies. The KNOX I subfamily comprised ZmTALE1, ZmTALE10–12, ZmTALE14, ZmTALE17, ZmTALE22, ZmTALE25, ZmTALE26, ZmTALE31, ZmTALE37, and ZmTALE38, whereas the KNOX II subfamily contained three members: ZmTALE18, ZmTALE28, and ZmTALE35. Notably, the KNOX III subfamily consisted exclusively of six maize TALE proteins (ZmTALE6, ZmTALE15, ZmTALE20, ZmTALE24, ZmTALE39, and ZmTALE40), with no homologs from the other analyzed plant species clustering within this clade. The BEL1-like subfamily was the largest, comprising the other 21 members (Figure 3). The topology of the ML tree was further supported by a neighbor-joining analysis, which showed highly similar clustering patterns (Supplementary Figure S2).

To further investigate the evolutionary mechanisms underlying the expansion of the maize TALE family, gene duplication events were systematically analyzed. All identified duplicated ZmTALE gene pairs were classified as segmental duplication events, whereas no tandem duplication pairs were detected, indicating that large-scale chromosomal duplication rather than local gene duplication contributed predominantly to the expansion of the ZmTALE family (Supplementary Table S2). Furthermore, Ka/Ks analysis of the syntenic ZmTALE gene pairs revealed that all Ka/Ks ratios were lower than 1 (Supplementary Table S2), suggesting that these duplicated genes have been subjected to strong purifying selection during evolution, which likely contributed to the maintenance of conserved TALE protein functions).

In addition, maize ZmTALE proteins generally exhibited closer evolutionary relationships with their homologs from the grass species rice and sorghum than with those from dicotyledonous plants. For example, ZmTALE23 formed a closely related sister clade with homologous proteins from sorghum and rice within the BEL1-like subfamily, whereas ZmTALE31 clustered closely with its rice homolog in the KNOX I subfamily, suggesting a high degree of evolutionary conservation among TALE genes within the Poaceae lineage.

### 2.4. Gene Structure and Conserved Motif Analysis of the ZmTALE Gene Family

**Figure 4:**
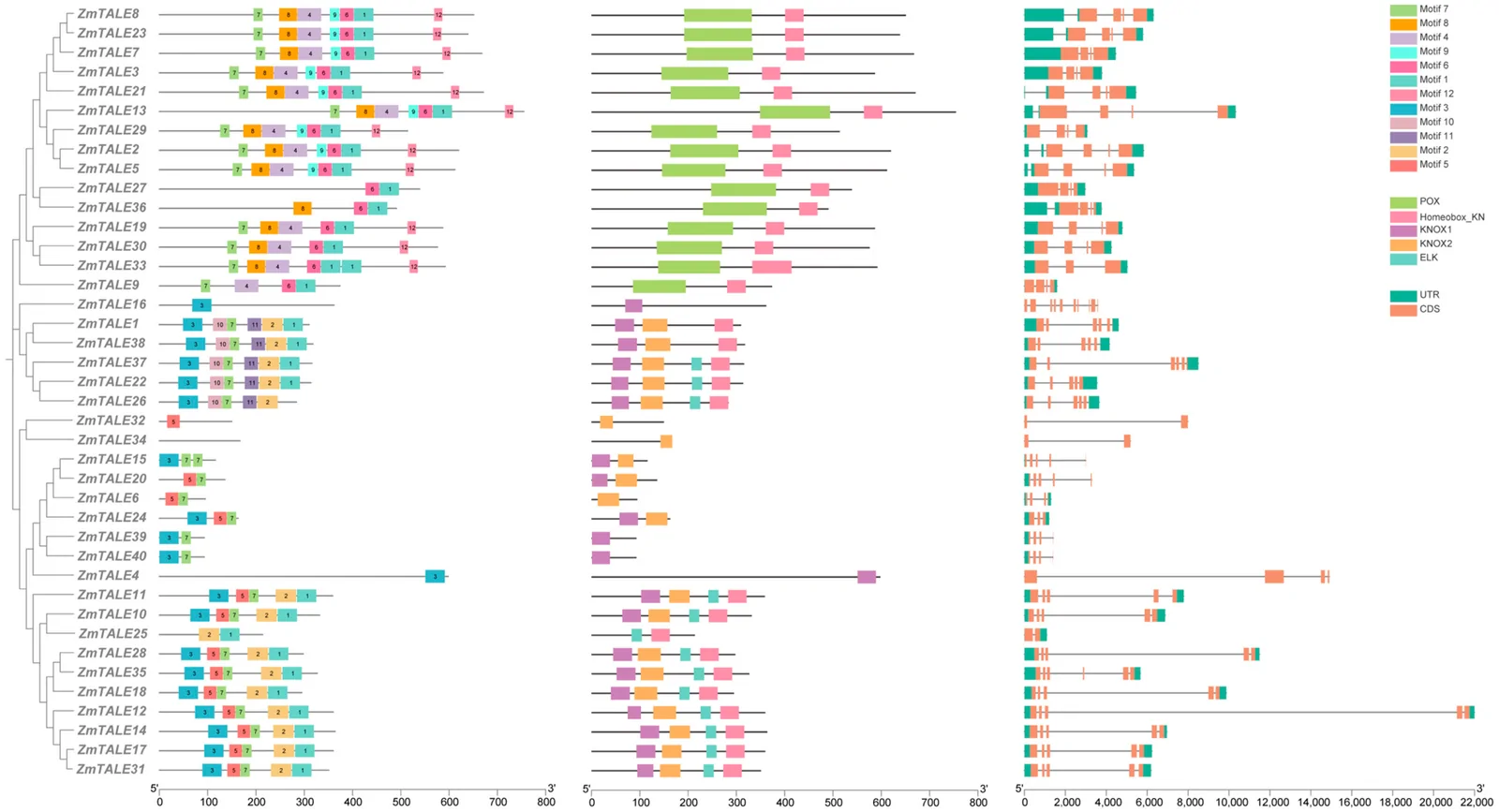
**Analysis of gene structures and conserved motifs of ZmTALE gene family.**

Gene structure analysis revealed considerable variation in exon-intron organization among *ZmTALE* genes. The number of exons ranged from 2 to 6, and gene lengths varied substantially among family members. Genes belonging to the BEL1-like subfamily generally exhibited relatively simple gene structures, containing 2–4 exons and gene lengths of less than 10 kb (Figure 4). In contrast, members of the KNOX subfamily displayed more complex exon-intron organizations, typically containing 4–6 exons. Some genes, such as ZmTALE31, exceeded 20 kb in length and possessed relatively long intronic regions. Members within the same subfamily exhibited highly similar exon-intron organization patterns, further supporting the reliability of the phylogenetic classification.

Conserved motif analysis using the MEME program identified 12 conserved motifs (Motifs 1–12) among the ZmTALE proteins. Members of the BEL1-like subfamily predominantly contained Motifs 7; 6; 4; 9; and 1; with highly conserved motif composition and arrangement within the subfamily (Figure 4). In contrast; proteins in the KNOX subfamily primarily shared Motifs 3; 10; 7; 2; and 1. Notably; Motif 1 and Motif 7 were widely conserved in both subfamilies; suggesting that these motifs may represent core structural features of TALE proteins.

### 2.5. Characterization of Cis-Acting Regulatory Element Within ZmTALE Promoters

**Figure 5:**
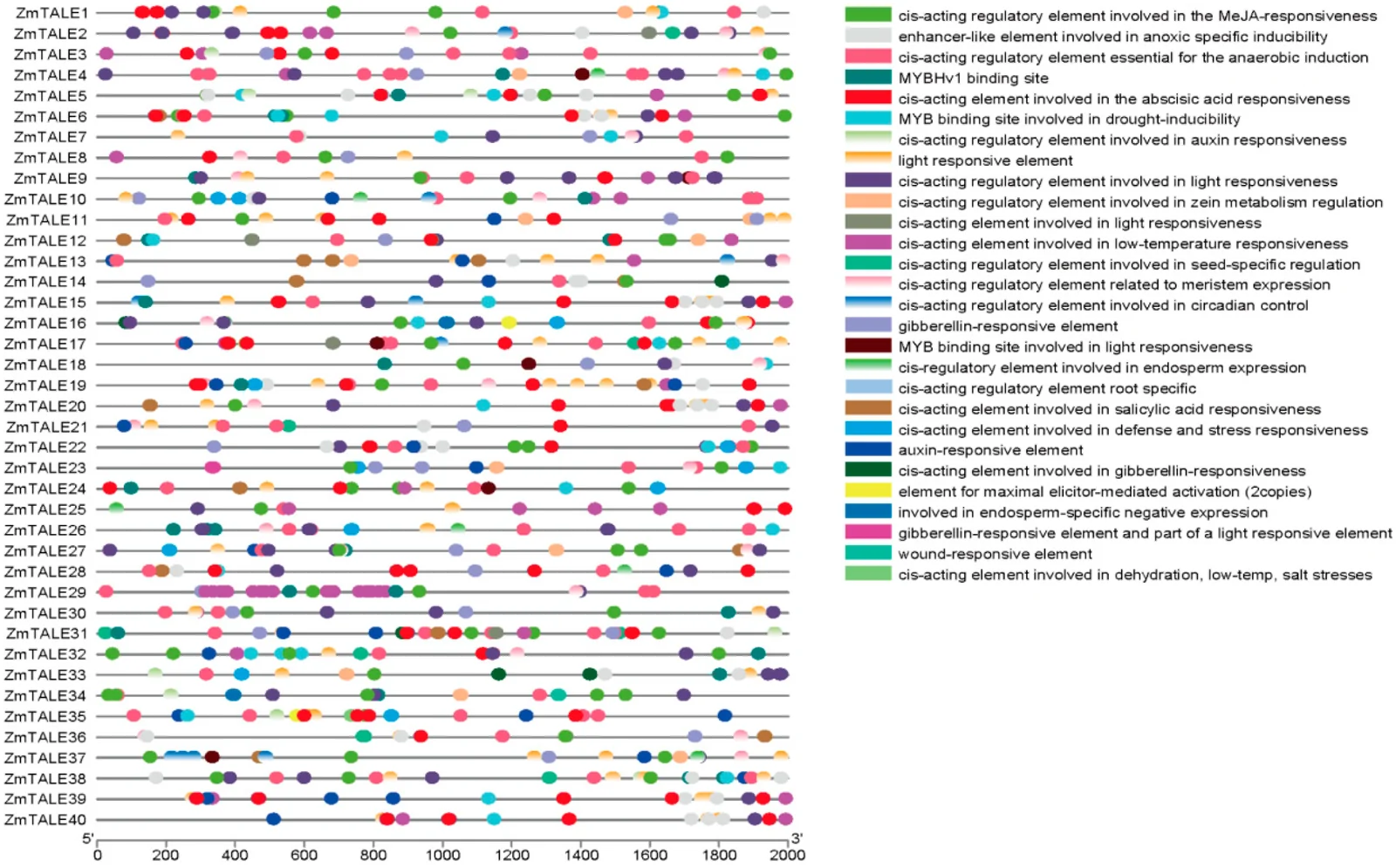
**Analysis of cis-acting elements in promoters of ZmTALE gene family.** The horizontal axis (0–2000 bp) represents the promoter region upstream of the transcription start site (TSS), with 2000 bp being the standard length for plant promoter analysis, and the vertical axis (ZmTALE1–40) represents the promoters of each family member.

To investigate the potential regulatory mechanisms of the ZmTALE gene family, the 2000-bp genomic sequences upstream of the transcription start site (TSS) of each ZmTALE gene were analyzed for cis-acting regulatory elements (CAREs) using the PlantCARE database. A total of more than 20 types of cis-acting regulatory elements were identified and classified into five major functional categories, including light-responsive, phytohormone-responsive, stress-responsive, tissue-specific, and growth- and development-related elements (Figure 5).

Among these elements, light-responsive elements were detected in the promoter regions of all 40 *ZmTALE* genes, representing the most abundant and widely distributed class of cis-elements, suggesting that light signaling may play a ubiquitous role in regulating the transcription of *ZmTALE* genes. With respect to phytohormone-responsive elements, abscisic acid-responsive elements (ABREs) and methyl jasmonate (MeJA)-responsive elements were identified in the promoter regions of the majority of ZmTALE genes (Figure 5). In addition, gibberellin-responsive elements (including GARE and P-box) and auxin-responsive elements (AuxREs) were detected in multiple family members. Salicylic acid-responsive elements (TCA-motifs) were also identified in the promoters of several *ZmTALE* genes.

Among the stress-responsive elements, anaerobic induction-responsive elements (AREs) were particularly enriched in the promoter regions of *ZmTALE3/5/8/10/15/20/23/29/31/33/36/38*. MYB-binding sites associated with drought inducibility were widely distributed throughout the ZmTALE promoter regions. In addition, defense- and stress-responsive elements, as well as dehydration-, low temperature-, and salt stress-responsive elements, together with wound-responsive elements, were identified in the promoters of several *ZmTALE* genes.

Regarding tissue-specific and developmental regulatory elements, meristem expression-related elements were detected in multiple ZmTALE promoters, consistent with the established roles of TALE transcription factors in meristem maintenance and development. Root-specific regulatory elements were identified in the promoters of *ZmTALE2/7/11/14/18/22/25/28/32/35*, suggesting that these genes may participate in root development or function (Figure 5). In addition, endosperm expression-related and seed-specific regulatory elements were identified in the promoters of several *ZmTALE* genes.

### 2.6. Synteny Analysis of the Maize TALE Gene Family

**Figure 6:**
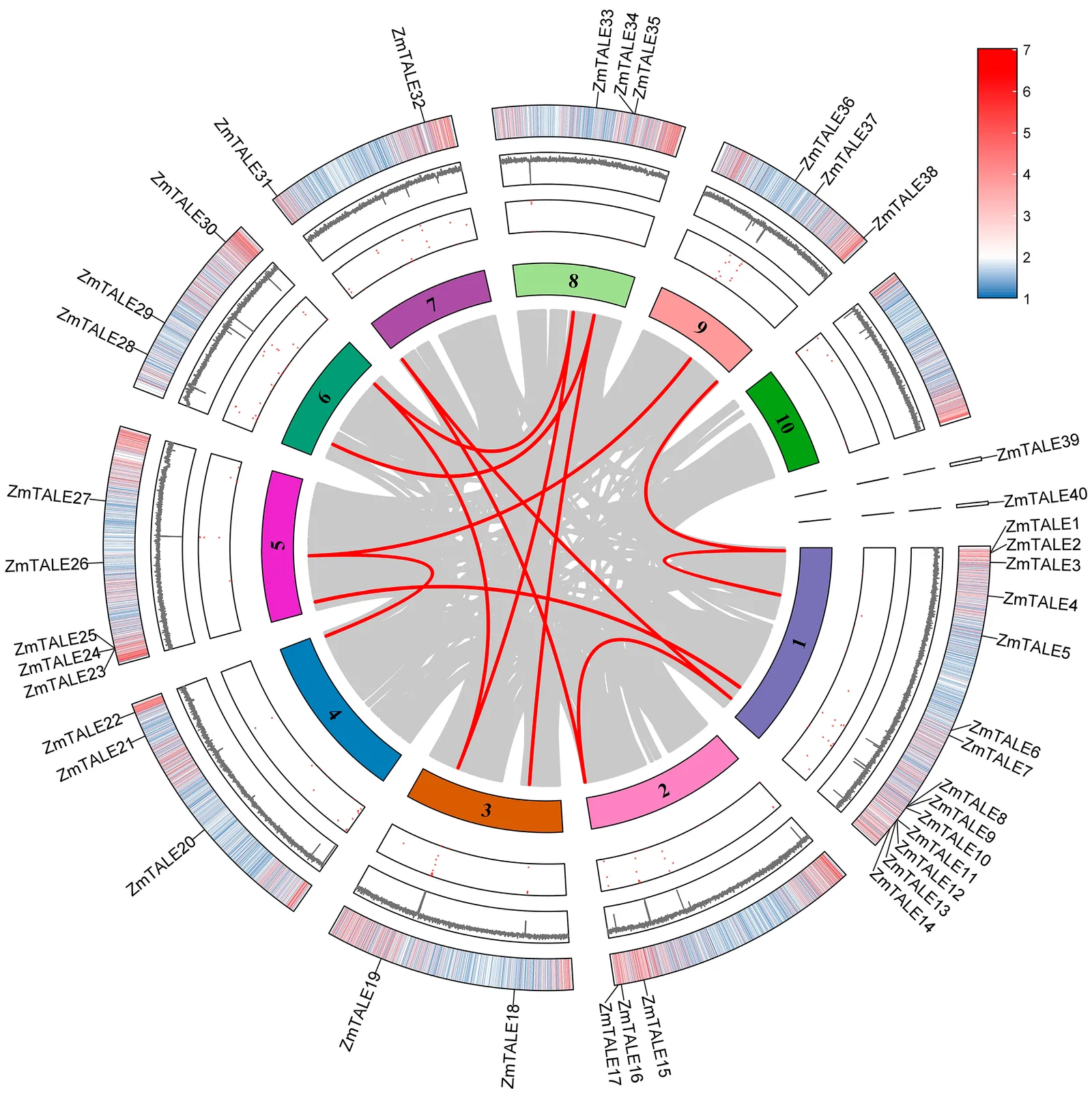
**Synteny analysis of TALE family genes in maize.** The gene names in black of outer circle correspond to the respective TALE loci. Gray lines in the inner circle indicate syntenic gene pairs, while bold red lines represent duplicated gene pairs within the maize TALE gene family. The number in the inner circle indicates the chromosome number.

To investigate the evolutionary mechanisms underlying the expansion of the ZmTALE gene family, an intraspecific synteny analysis was performed for the 40 *ZmTALE* genes, and the syntenic relationships were visualized using a Circos plot (Figure 6). Multiple syntenic gene pairs were identified among chromosomes 1, 5, 8, and 9, indicating extensive collinearity among these chromosomal regions. In particular, several *ZmTALE* genes located on chromosome 1 exhibited syntenic relationships with homologous genes on chromosomes 5, 8, and 9, suggesting that these genes originated from segmental duplication events. In addition, syntenic gene pairs were also observed between chromosomes 5, 8, and 9, implying the presence of conserved collinear blocks among these chromosomes (Figure 6). In contrast, few or no syntenic gene pairs were detected on chromosomes 2, 3, 4, 6, and 7, indicating that *ZmTALE* genes located on these chromosomes have not undergone extensive segmental duplication.

Overall, the synteny analysis demonstrated that segmental duplication represents the predominant mechanism underlying the expansion of the ZmTALE gene family. Most duplication events were distributed among chromosomes 1, 5, 8, and 9, consistent with the evolutionary history of maize, which has experienced ancient whole-genome duplication (WGD) events (Figure 6). Prominent collinear relationships were observed between the *ZmTALE* gene cluster on chromosome 1 and corresponding genes on chromosome 5 (including ZmTALE23–ZmTALE27), chromosome 8 (ZmTALE33–ZmTALE38), and chromosome 9 (ZmTALE36–ZmTALE37), further supporting the important contribution of segmental duplication to the expansion of the ZmTALE gene family.

**Figure 7:**
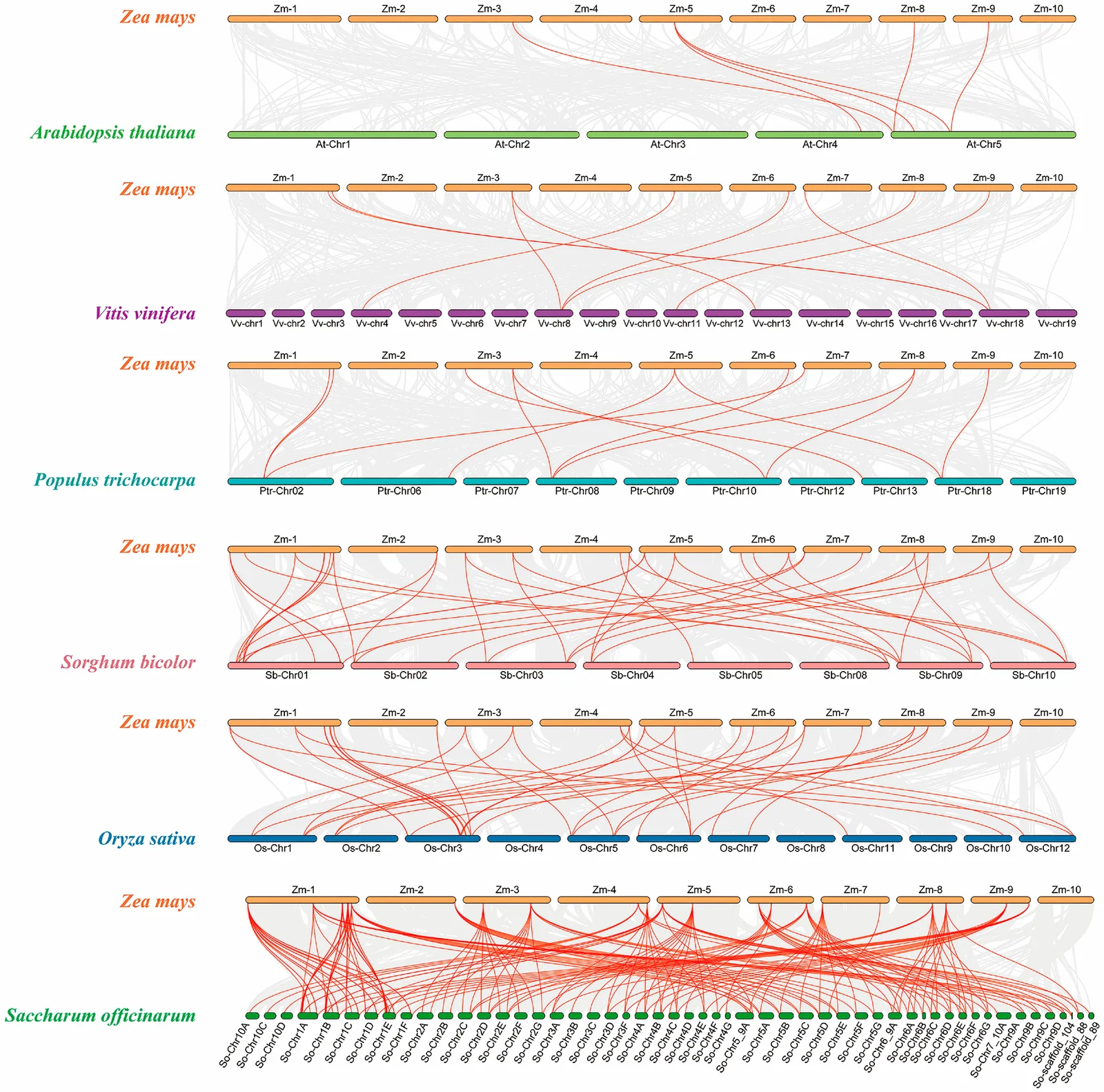
**Synteny analysis of TALE family genes between maize and other plant species.** Gray lines indicate syntenic gene pairs between maize and *Arabidopsis thaliana* (*Oryza sativa*, *Sorghum bicolor*, *Saccharum officinarum*, *Vitis vinifera*, or *Populus trichocarpa*), while bold red lines represent co-syntenic gene pairs of the TALE gene family between maize and other plant species.

Comparative synteny analysis was further performed between maize (*Zea mays*) and six representative plant species in phylogenetic analysis. The results revealed distinct levels of syntenic conservation among species. Relatively few orthologous gene pairs were identified between maize and the dicotyledonous species, including *A. thaliana* (6 gene pairs), *V. vinifera* (9 gene pairs), and *P. trichocarpa* (12 gene pairs) (Figure 7). In contrast, substantially stronger syntenic relationships were observed between maize and the monocotyledonous grasses, with 34 orthologous gene pairs detected between *ZmTALE* and *OsTALE*, and 36 between *ZmTALE* and *SbTALE* (Figure 7). The highest level of collinearity was identified between *ZmTALE* and *SoTALE* (*S. officinarum* TALE genes).

Overall, the comparative synteny analysis demonstrated that *ZmTALE* genes exhibit relatively limited syntenic conservation with the dicotyledonous species *A. thaliana*, *V. vinifera*, and *P. trichocarpa*, whereas markedly higher levels of collinearity were observed with monocotyledonous species, particularly members of the Poaceae family. Notably, sugarcane shared the greatest number of orthologous TALE gene pairs with maize, indicating a high degree of genomic conservation and supporting the close evolutionary relationship between these two species. These findings further suggest that the TALE gene families of maize and sugarcane have been highly conserved during evolution and likely originated from a common ancestral lineage.

### 2.7. Phenotypic and Physiological Responses of Three Maize Inbred Lines to Low-Phosphorus Stress

**Figure 8:**
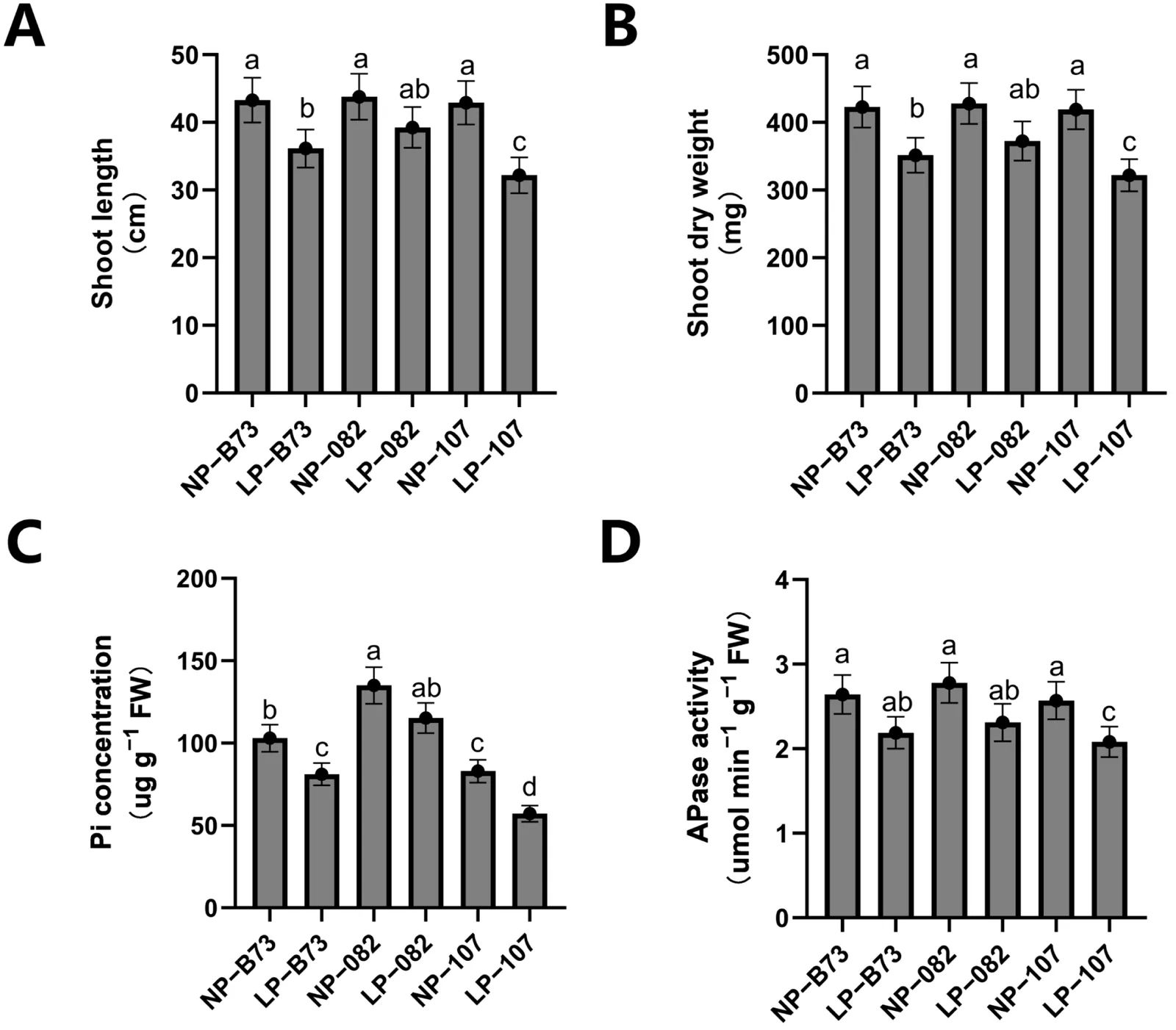
**Phenotypic and physiological responses of three maize inbred lines under 14 days of low-phosphorus treatment.** Statistical analysis of shoot length (**A**), shoot dry weight (**B**), inorganic Pi concentration (**C**), and acid phosphatase activity (**D**) under normal and low Pi conditions. Different letters above bars represent statistically significant differences between groups (*p* < 0.05) as determined by two-way ANOVA followed by the LSD test and Waller–Duncan’s test.

To investigate the physiological responses of maize to phosphorus deficiency and identify inbred lines with contrasting phosphorus-use efficiencies, three maize inbred lines (082, Ye107, and B73) were subjected to low-phosphorus (LP; 5 μM KH_2_PO_4_) treatment, while seedlings grown in complete nutrient solution served as the normal-phosphorus (NP) controls. After 14 days of treatment, shoot length, shoot dry weight, leaf inorganic phosphate (Pi) content, and acid phosphatase (APA) activity were determined (Figure 8).

Under NP conditions, no significant differences were observed among the three inbred lines in terms of shoot length, shoot dry weight, or APA activity. In contrast, significant variation in leaf Pi content was detected, with inbred line 082 exhibiting the highest Pi concentration, followed by B73, whereas Ye107 accumulated the lowest Pi level (Figure 8).

LP treatment markedly inhibited plant growth in all three maize inbred lines. Both shoot length and shoot dry weight were significantly reduced following phosphorus deprivation, although the extent of inhibition differed among genotypes. The greatest reductions were observed in Ye107, followed by B73, whereas 082 exhibited the smallest decrease in both traits, indicating greater tolerance to phosphorus deficiency (Figure 8A,B).

LP treatment caused a substantial decline in Pi accumulation in all three inbred lines, and leaf Pi concentration was even more severely affected by phosphorus deficiency than the other physiological traits (Figure 8C). Similarly, APA activity decreased significantly under LP treatment in all three inbred lines. The reduction was most pronounced in Ye107, while 082 and B73 exhibited comparable decreases in enzyme activity (Figure 8D), with a response pattern consistent with those observed for shoot growth and Pi concentration.

Overall, the physiological and biochemical analyses demonstrated clear genotypic differences in the responses of maize to phosphorus deficiency. Among the three inbred lines, 082 exhibited the highest tolerance to LP stress, B73 showed intermediate tolerance, and Ye107 was the most sensitive, indicating substantial natural variation in phosphorus-use efficiency.

### 2.8. Expression Responses of the ZmTALE Gene Family to Low-Phosphorus Stress

**Figure 9:**
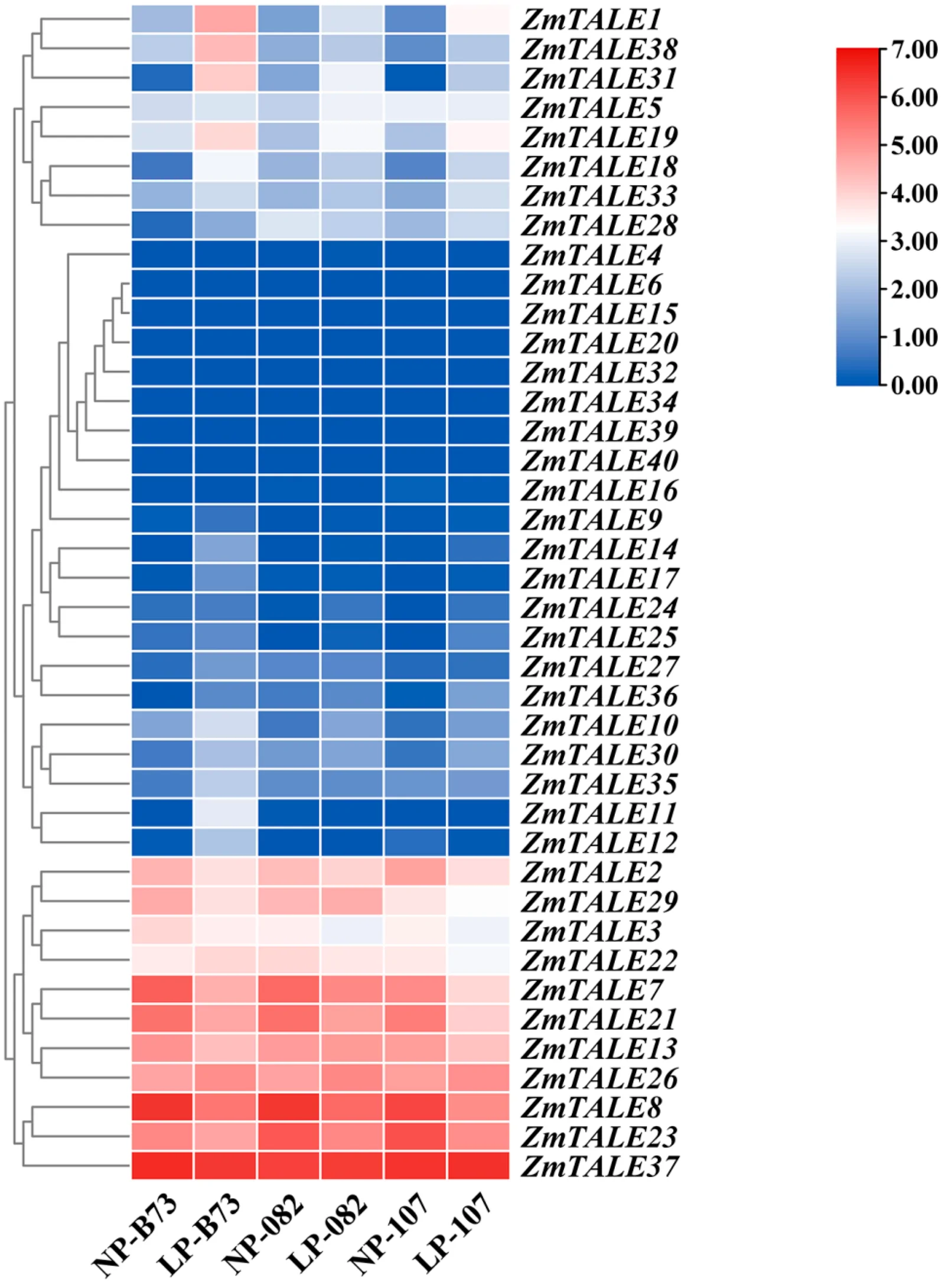
**Heatmap showing the expression level of ZmTALEs in three maize inbred lines under different phosphorus treatments.** The (FPKM + 1) values are log2-based. Red and blue indicate high and low expression levels, respectively. Each sample had three biological replicates.

Hence, to investigate the transcriptional responses of the ZmTALE gene family to low-phosphorus (LP) stress, RNA-seq datasets generated from three maize inbred lines (B73, 082, and Ye107) under normal-phosphorus (NP) and low-phosphorus (LP) treatments of 14 days were analyzed. Hierarchical clustering analysis of the expression profiles of the 40 *ZmTALE* genes revealed substantial variation among different phosphorus treatments and genetic backgrounds. Based on their expression patterns, the ZmTALE genes could be broadly classified into three groups (Figure 9). The first group comprised genes that exhibited relatively low expression under NP conditions but were markedly induced or repressed following LP treatment in all three inbred lines. The second group consisted of genes showing constitutively low or nearly undetectable expression, with little or no response to LP stress. The third group displayed genotype-dependent expression patterns, with some genes being induced whereas others were repressed under LP treatment depending on the genetic background (Figure 9).

Several ZmTALE genes, including *ZmTALE1*, *ZmTALE18*, *ZmTALE19*, *ZmTALE28*, *ZmTALE31*, *ZmTALE33*, and *ZmTALE38*, showed increased transcript abundance in B73, 082, and Ye107 following LP treatment based on RNA-seq analysis, suggesting that these genes may be responsive to phosphorus deficiency. In contrast, *ZmTALE4*, *ZmTALE6*, *ZmTALE9*, *ZmTALE15*, *ZmTALE16*, *ZmTALE20*, *ZmTALE32*, *ZmTALE34*, *ZmTALE39*, and *ZmTALE40* exhibited relatively stable or low transcript levels under both NP and LP conditions, indicating limited transcriptional responses to LP stress. In addition, several genes, including *ZmTALE2*, *ZmTALE3*, *ZmTALE7*, *ZmTALE8*, *ZmTALE13*, *ZmTALE21*, *ZmTALE22*, *ZmTALE23*, *ZmTALE26*, *ZmTALE29*, and *ZmTALE37*, maintained relatively high expression levels under both conditions but displayed variation among different maize inbred lines, suggesting genotype-dependent transcriptional regulation (Figure 9).

Comparative analysis among the three maize inbred lines revealed that B73 exhibited a greater number of differentially expressed *ZmTALE* genes under LP conditions than 082 and Ye107. These results suggest that *ZmTALE* transcriptional responses to phosphorus deficiency vary among maize genetic backgrounds, with potential differences in regulatory sensitivity among inbred lines.

**Figure 10:**
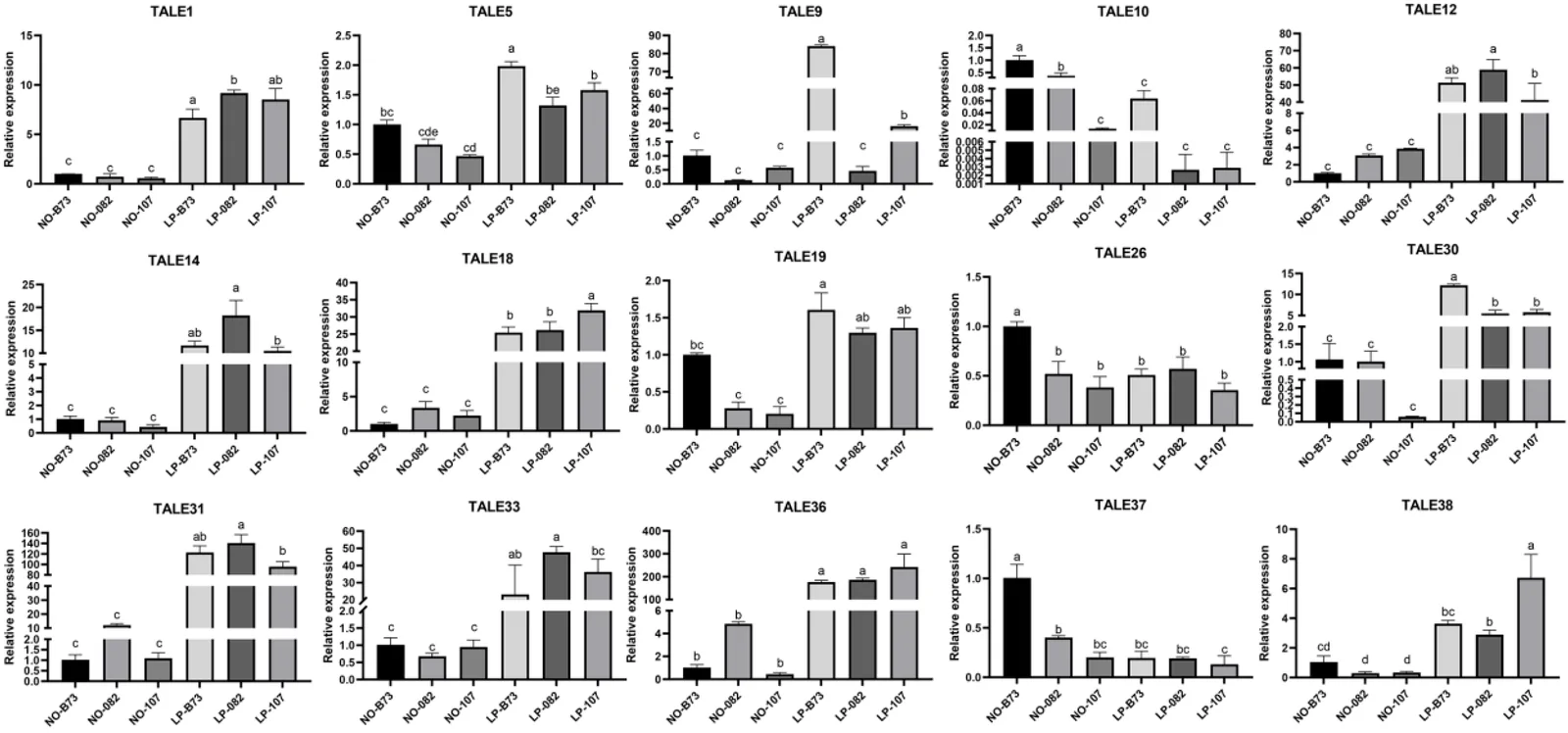
**Relative expression levels of maize TALE genes with qRT-PCR analysis.** No refers to maize seedling samples grown under normal culture conditions, and LP refers to seedlings grown under low-phosphorus conditions. Different letters above bars represent statistically significant differences between groups (*p* < 0.05) as determined by two-way ANOVA followed by the LSD test and Waller–Duncan’s test.

To further evaluate the reliability of the RNA-seq results, 15 representative ZmTALE genes were selected for RT-qPCR validation based on their differential expression patterns, expression abundance, and potential responsiveness to LP stress. The RT-qPCR analysis confirmed that several RNA-seq-identified LP-responsive genes, including *ZmTALE1*, *ZmTALE18*, *ZmTALE31*, and *ZmTALE33*, exhibited similar expression trends in all three maize inbred lines (Figure 10). In addition, *ZmTALE5*, *ZmTALE12*, *ZmTALE14*, *ZmTALE30*, and *ZmTALE36* showed significant induction under LP conditions, whereas *ZmTALE10* and *ZmTALE37* were downregulated following LP treatment. However, some differences were observed between RNA-seq and RT-qPCR results, likely due to differences in detection sensitivity, normalization procedures, and experimental platforms.

Because the M73 genome is highly similar to the B73 reference genome, the B73 genome annotation can be reliably applied for transcriptome analysis and functional annotation of M73. In addition, our observations and preliminary experiments indicated that M73 exhibits a more sensitive transcriptional response to short-term phosphorus deficiency compared with B73. Therefore, to further refine the identification of ZmTALE genes potentially involved in the early response to phosphorus deficiency and to facilitate subsequent functional characterization, an additional short-term LP treatment was performed using the maize inbred line M73. Whole seedlings were sampled at 0, 0.5, 1, 3, 6, and 12 h after LP treatment, followed by RNA-seq analysis to characterize the temporal expression profiles of the ZmTALE gene family.

**Figure 11:**
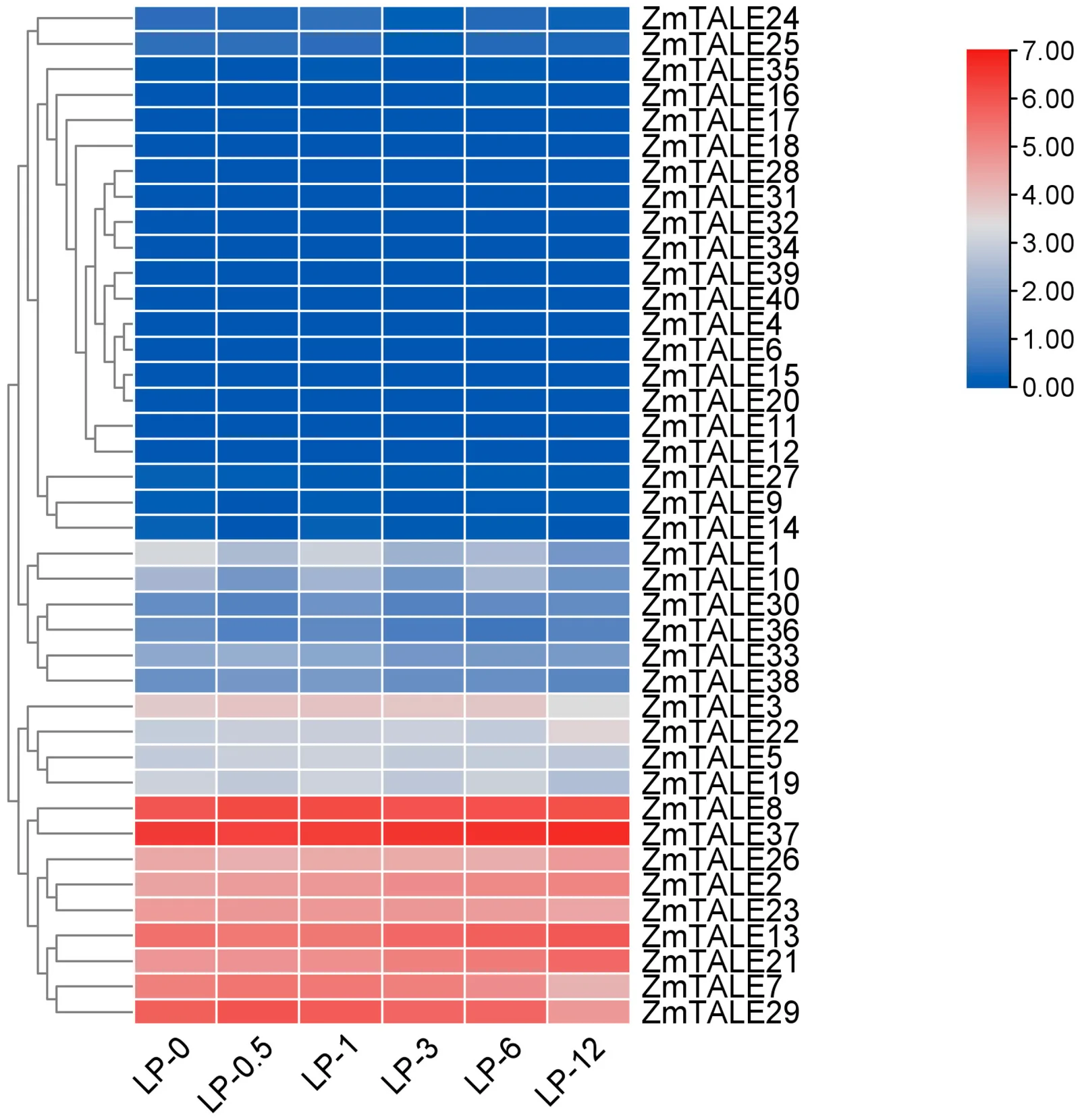
**Heatmap showing the expression level of ZmTALEs in M73 inbred line under short-term LP treatment (0, 0.5, 1, 3, 6, and 12 h).** The (FPKM + 1) values are log2-based. Red and blue indicate high and low expression levels, respectively. Each sample had three biological replicates.

The expression patterns observed during the short-term LP treatment were generally consistent with those obtained from the 14-day long-term experiment. A total of 20 ZmTALE genes, including *ZmTALE4*, *ZmTALE6*, *ZmTALE9–12*, *ZmTALE14–18*, *ZmTALE20*, *ZmTALE24*, *ZmTALE25*, *ZmTALE27*, *ZmTALE28*, *ZmTALE31*, *ZmTALE32*, *ZmTALE35*, *ZmTALE39*, and *ZmTALE40*, exhibited little or no transcriptional response to LP treatment throughout the time course. In contrast, *ZmTALE2*, *ZmTALE7*, *ZmTALE8*, *ZmTALE13*, *ZmTALE21*, *ZmTALE23*, *ZmTALE26*, *ZmTALE29*, and *ZmTALE37* maintained relatively high transcript abundance under both control and LP conditions, indicating constitutively high expression irrespective of phosphorus availability (Figure 11). The remaining *ZmTALE* genes displayed diverse temporal expression patterns following LP treatment, suggesting differential transcriptional regulation during the early stages of phosphorus deficiency.

### 2.9. ZmTALE10, ZmTALE14, and ZmTALE31 Are Nuclear-Localized Transcriptional Activators

Based on the integrated transcriptomic and RT–qPCR analyses presented in Section 2.7 and Section 2.8, ZmTALE10, ZmTALE14, and ZmTALE31 were identified as key candidate transcription factors responsive to low-phosphorus stress. To further verify their molecular characteristics and gain insight into their potential regulatory roles, subcellular localization and transcriptional activation assays were subsequently performed.

**Figure 12:**
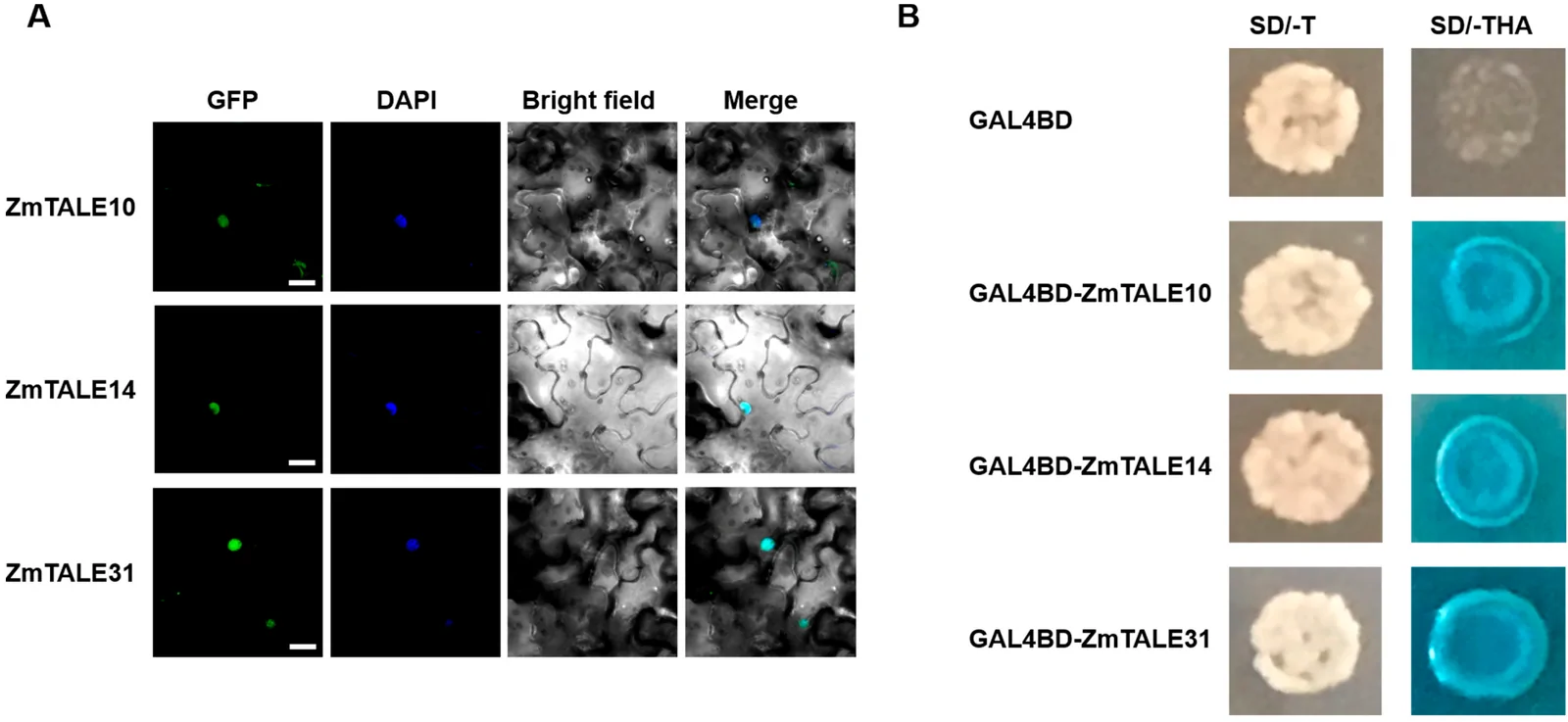
**Subcellular localization and transcriptional activity analysis of ZmTALE10/14/31.** (**A**) Confocal images of localization of GFP-ZmTALE10/14/31 in tobacco leaf epidermal cells. DAPI (40,6-Diamidino-2-phenylindole dihydrochloride), a nuclear staining dye. Scale bars = 10 µm. (**B**) The transcriptional activity assays in yeast cells. The yeast carrying GAL4BD-ZmTALE10/14/31 grew on a medium without Trp, His, and Ade (SD/–THA) and induced the expression activity of X-α-Gal, while the negative control was not.

To investigate the subcellular localization of ZmTALE proteins, the full-length coding sequences of ZmTALE10, ZmTALE14, and ZmTALE31 were fused to GFP and transiently expressed in *Nicotiana benthamiana* leaf epidermal cells. As shown in Figure 12A, the GFP fluorescence signals of all three fusion proteins were exclusively localized to the nucleus, whereas the control GFP protein was distributed throughout both the nucleus and cytoplasm. These results demonstrate that ZmTALE10, ZmTALE14, and ZmTALE31 are nuclear-localized proteins, consistent with their predicted roles as transcription factors.

To determine whether these ZmTALE proteins possess transcriptional activation activity, yeast transcriptional activation assays were performed using the GAL4-based system. Yeast cells expressing BD-ZmTALE10, BD-ZmTALE14, or BD-ZmTALE31 grew normally on selective SD/-Trp/-His/-Ade medium and produced blue colonies in the presence of X-α-Gal, whereas yeast cells transformed with the empty pGBKT7 vector failed to grow or produce blue coloration (Figure 12B). These results indicate that ZmTALE10, ZmTALE14, and ZmTALE31 possess intrinsic transcriptional activation activity in yeast, suggesting that they function as nuclear-localized transcriptional activators.

## 3. Discussion

TALE TFs constitute one of the most evolutionarily conserved homeobox protein families in plants and are widely recognized as central regulators of meristem maintenance, organogenesis, hormone homeostasis, and environmental adaptation [1,4,6]. Although genome-wide analyses of TALE genes have recently been reported in *Arabidopsis* [7], soybean [25], sorghum [26], and cucumber [27] and several other species, systematic studies in maize, particularly those focusing on phosphorus-deficiency responses, remain limited. In the present study, we integrated genome-wide identification, evolutionary analysis, transcriptome profiling, physiological characterization, and preliminary functional validation to provide a comprehensive overview of the maize TALE gene family. Our findings not only reveal the evolutionary characteristics of *ZmTALE* genes but also provide experimental evidence supporting their potential involvement in adaptation to phosphorus deficiency.

The identification of 40 ZmTALE genes and their classification into the BEL1-like, KNOX I, KNOX II, and KNOX III subfamilies agree well with previous reports [28] indicating that the overall evolutionary framework of TALE proteins has been highly conserved throughout land plant evolution. Similar exon–intron organizations, conserved motif compositions, and highly comparable three-dimensional structures among members of the same subfamily further support the existence of strong purifying selection acting on TALE proteins. Interestingly, comparative phylogenetic analysis demonstrated that the KNOX III clade was almost exclusively composed of monocotyledonous species [29], suggesting that this subgroup may have undergone lineage-specific retention or functional specialization after the divergence of monocots and dicots. Moreover, segmental duplication rather than tandem duplication represented the major driving force underlying ZmTALE family expansion, which is consistent with the ancient whole-genome duplication history of maize. The stronger syntenic relationships observed between maize and other Poaceae species [30], particularly sugarcane and sorghum, further indicate that most TALE genes originated before grass diversification and subsequently experienced lineage-specific evolution [4].

Increasing evidence indicates that TALE proteins function not only as developmental regulators but also as important integrators of phytohormone signaling and abiotic stress responses. Consistent with these observations, promoter analysis revealed that the majority of ZmTALE genes contain abundant cis-regulatory elements associated with abscisic acid, methyl jasmonate, auxin, gibberellin, and drought responses, together with numerous root-specific and meristem-related regulatory elements. Previous studies have demonstrated that KNOX proteins maintain shoot apical meristem activity through promoting cytokinin biosynthesis [31] while repressing gibberellin accumulation [32], whereas BEL1-like proteins cooperate with KNOX proteins to regulate organ initiation, vascular differentiation, and root patterning [33]. Therefore, the coexistence of multiple hormone-responsive elements in ZmTALE promoters strongly suggests that maize TALE proteins may integrate developmental programs with environmental cues through extensive hormone crosstalk. Considering that root architecture is one of the most important determinants of phosphorus acquisition efficiency [34,35], the enrichment of root-specific regulatory elements further implies that TALE proteins may contribute to phosphorus adaptation indirectly by regulating root system development rather than directly controlling phosphate transporter genes.

The physiological analyses conducted in this study further support the existence of substantial natural variation in phosphorus-use efficiency among maize germplasms. Although the three inbred lines displayed similar growth under sufficient phosphorus conditions, phosphorus deficiency caused markedly different reductions in shoot growth, biomass accumulation, acid phosphatase activity, and inorganic phosphate content, with 082 exhibiting the highest tolerance and Ye107 showing the greatest sensitivity. These contrasting phenotypes provided an excellent biological basis for subsequent transcriptome analyses and enabled the identification of genotype-dependent LP-responsive TALE genes. Interestingly, RNA-seq combined with RT–qPCR validation identified several genes, including *ZmTALE1*, *ZmTALE5*, *ZmTALE12*, *ZmTALE14*, *ZmTALE18*, *ZmTALE30*, *ZmTALE31*, *ZmTALE33*, and *ZmTALE36*, that were consistently induced by phosphorus deficiency across different genetic backgrounds, whereas ZmTALE10 and ZmTALE37 were significantly repressed. These expression patterns suggest that different TALE members have undergone considerable functional divergence during evolution, with some functioning in developmental regulation and others participating in environmental adaptation. Furthermore, the genotype-dependent transcriptional responses observed among B73, 082, and Ye107 indicate that allelic variation may substantially influence TALE-mediated phosphorus-deficiency responses and therefore represents valuable genetic resources for improving phosphorus-use efficiency through molecular breeding.

A notable advance of the present study beyond previous genome-wide analyses is the preliminary functional characterization of three representative LP-responsive TALE proteins. Subcellular localization assays demonstrated that ZmTALE10, ZmTALE14, and ZmTALE31 are exclusively localized in the nucleus, whereas yeast transcriptional activation assays confirmed that all three proteins possess transcriptional activation activity. These experimental results provide direct evidence that these LP-responsive TALE proteins function as nuclear transcription factors, supporting the predictions derived from sequence analysis.

Interestingly, the three genes displayed distinct transcriptional responses to low-phosphorus stress. *ZmTALE14 and ZmTALE31* were markedly induced, whereas *ZmTALE10* was transcriptionally repressed, suggesting that different members of the maize TALE family may participate in low-phosphorus responses through distinct regulatory mechanisms. Similar expression divergence has been reported for TALE family members in other plant species, indicating that functional diversification is a common feature of this transcription factor family. However, the biological significance of these contrasting expression patterns and their downstream regulatory targets remain to be determined.

Although our results demonstrate that several ZmTALE genes are responsive to phosphorus deficiency and encode functional nuclear transcription factors, their precise roles in phosphorus-deficiency adaptation have not yet been established. The present study provides a foundation for future functional investigations, including gene knockout or overexpression analyses, identification of downstream target genes, and elucidation of the molecular pathways regulated by individual ZmTALE proteins under phosphorus-deficient conditions.

## 4. Materials and Methods

### 4.1. Identification of the TALE Gene Family in Zea mays

Putative TALE family members in maize were initially retrieved from the Plant Transcription Factor Database (PlantTFDB v5.0) [36] based on the TALE TF annotation, and their corresponding protein sequences were downloaded. To ensure comprehensive identification, these maize TALE proteins were subsequently used as queries to perform BLASTP searches against the JGI Phytozome 13 database, and all homologous protein sequences were collected.

In addition, the amino acid sequences of the 22 previously reported *Arabidopsis* TALE proteins (*A. thaliana* TAIR10) were downloaded from the JGI Phytozome 13 database and used as reference sequences. A local BLASTP search was then performed against the maize reference genome (*Zea mays* B73 RefGen_v5; Zm-B73-REFERENCE-NAM-5.0.55) using TBtools II [37], and candidate TALE proteins were extracted according to the obtained gene IDs. Redundant sequences identified by the two independent approaches were removed to generate a non-redundant candidate gene set. The overlap between the two identification strategies was visualized using a Venn diagram generated with TBtools II.

To further verify the authenticity of the candidate TALE proteins, the presence of the conserved TALE homeodomain was examined using the NCBI Batch Conserved Domain Search (Batch CD-Search) with an E-value threshold of 0.01, together with the SMART database. Candidate proteins lacking the characteristic TALE conserved domain were discarded. The remaining non-redundant proteins containing the complete TALE conserved domain were ultimately designated as members of the maize TALE gene family.

### 4.2. Physicochemical Properties and Structural Characterization of ZmTALE Proteins

The genomic information of the identified ZmTALE genes, including gene accession numbers, chromosomal locations, genomic coordinates, coding sequence (CDS) lengths, and strand orientation, was retrieved from the Phytozome database. The presence of N-terminal signal peptides was predicted using the SignalP 5.0 online server [38]. The physicochemical properties of the predicted ZmTALE proteins, including amino acid length, molecular weight (MW), theoretical isoelectric point (pI), instability index (II), aliphatic index (AI), and grand average of hydropathicity (GRAVY), were calculated using the ExPASy ProtParam module [39] implemented in TBtools II. Three-dimensional (3D) protein structure models were generated using the SWISS-MODEL server based on homology modeling [40]. The subcellular localization of ZmTALE proteins was predicted using Plant-mPLoc [41].

### 4.3. Phylogenetic Analysis and Classification of the ZmTALE Gene Family

To investigate the evolutionary relationships and classify the ZmTALE proteins into distinct subfamilies, the TALE protein sequences of *A. thaliana*, *O. sativa*, *S. bicolor*, *S. officinarum*, *P. trichocarpa*, and *V. vinifera* were downloaded from the Phytozome database and used as reference sequences. Multiple sequence alignment of TALE proteins from maize, *A. thaliana*, *O. sativa*, and *V. vinifera* was performed using MEGA 12 [42]. Based on the multiple sequence alignment results, a phylogenetic tree was constructed using the maximum likelihood (ML) method implemented in MEGA 12. The Poisson substitution model was applied with partial deletion of gaps and missing data (site coverage cutoff = 95%). The reliability of the inferred phylogenetic relationships was evaluated by performing 1000 bootstrap replicates, and the resulting bootstrap values were used to assess branch support. The resulting phylogenetic tree was subsequently visualized and annotated using the online tool EvolView (v2024) [43].

### 4.4. Gene Structure, Conserved Motif, and Protein Structure Analyses of the ZmTALE Gene Family

Gene structure information, including the distribution of exons, introns, and untranslated regions (UTRs), was extracted from the maize genome annotation file to characterize the exon-ntron organization of *ZmTALE* genes. The gene structures and conserved motif distributions were visualized using TBtools II [37].

To further investigate the conserved motif composition of ZmTALE proteins, motif analysis was performed using the Multiple Expectation Maximization for Motif Elicitation (MEME) online server [44]. The maximum number of motifs was set to 12, with motif widths ranging from 6 to 50 amino acid residues, while all other parameters were maintained at their default settings.

### 4.5. Chromosomal Location and Synteny Analysis of the Maize TALE Gene Family

The chromosomal distribution of *ZmTALE* genes was determined based on the maize genome annotation file obtained from the Phytozome database. Information on chromosome lengths and the genomic coordinates (start and end positions) of each *ZmTALE* gene was extracted from the annotation file. The chromosomal locations of *ZmTALE* genes were visualized using the Gene Location Visualize function implemented in TBtools II.

To investigate the evolutionary mechanisms underlying the expansion of the ZmTALE gene family, intraspecific synteny analysis was performed using the MCScanX [45] module integrated into TBtools II. Segmental and tandem duplication events were identified and visualized to evaluate their respective contributions to the expansion of the ZmTALE gene family within the maize genome. To further explore the evolutionary conservation of *TALE* genes among different plant species, interspecific synteny analysis was conducted between maize and representative monocotyledonous and dicotyledonous species, including *A. thaliana*, *O. sativa*, *V. vinifera*, and other selected species. Conserved collinear gene pairs were identified using the MCScanX algorithm implemented in TBtools, and the resulting syntenic relationships were visualized to infer the evolutionary conservation and divergence of the TALE gene family during plant evolution.

### 4.6. Cis-Acting Regulatory Element Analysis of ZmTALE Gene Promoters

To investigate the potential regulatory mechanisms of *ZmTALE* genes, the 2000-bp genomic sequences upstream of the transcription start site (TSS) of each ZmTALE gene were extracted from the maize genome using TBtools and defined as the putative promoter regions. The promoter sequences were subsequently submitted to the PlantCARE database [46] to identify putative cis-acting regulatory elements (CAREs).

The identified cis-acting elements were categorized according to their predicted biological functions, with particular emphasis on elements associated with light responsiveness, phytohormone responsiveness (including abscisic acid (ABA), gibberellin (GA), auxin (IAA), methyl jasmonate (MeJA), and salicylic acid (SA), abiotic stress responsiveness, and tissue-specific expression. The distribution and composition of cis-acting regulatory elements within the promoter regions of ZmTALE genes were visualized using TBtools.

### 4.7. Expression Profiling of ZmTALE Genes Under Low-Phosphorus Stress and RT-qPCR Validation

All plant materials are from the Maize Research Institute of Southwest University. Seeds of maize inbred lines were used for germination experiments under controlled conditions (growth chamber at 25 °C). The seeds were obtained from the Maize Research Institute of Southwest University (Beibei, Chongqing, China). We germinated the seeds on filter paper moistened with demineralized water. When the seedlings reached the three-leaf stage, uniformly growing plants were selected, roots were gently rinsed, and the seedlings were transferred to Hoagland nutrient solution (Coolaber Technology Co., Ltd., Beijing, China). 5 uM KH2PO4 was used for low-phosphorus treatment, and 5 mM KNO3 was used for high-nitrogen treatment. The plants were grown in a growth chamber with a photoperiod of 14 h light/10 h dark, day/night temperatures of 28 °C/24 °C, and 65% relative humidity. For each treatment, more than five healthy plants were pooled as a biological replicate, and three biological replicates were collected per condition.

To investigate the transcriptional responses of *ZmTALE* genes to low-phosphorus (LP) stress, transcriptome sequencing was performed using four maize inbred lines subjected to LP treatment. For the long-term LP treatment, three maize inbred lines (082, Ye107, and B73) were grown hydroponically in a nutrient solution containing 5 μM KH_2_PO_4_ from the V3 stage. After 14 days of LP treatment, whole seedlings were harvested for total RNA extraction. For the short-term LP treatment, the maize inbred line M73 was grown under the same hydroponic conditions, and whole seedlings were collected at 0, 0.5, 1, 3, 6, and 12 h after LP treatment for total RNA extraction. Plants grown in a complete nutrient solution under identical conditions were used as the corresponding controls.

For each sample, approximately 0.5 μg of high-quality total RNA was used for RNA sequencing (RNA-seq) library construction. Three independent biological replicates were included for each treatment and genotype. After RNA integrity was confirmed (RNA Integrity Number, RIN ≥ 7.0), sequencing libraries were constructed using a standard mRNA library preparation kit following the manufacturer’s instructions and sequenced on the Illumina NovaSeq 6000 platform to generate 150-bp paired-end reads by Biomarker Technologies Co., Ltd. (Beijing, China).

Raw sequencing reads were subjected to quality control by removing adaptor sequences, reads containing more than 10% ambiguous nucleotides, and low-quality reads with a high proportion of bases having a Phred quality score below 20. The resulting clean reads were mapped to the maize B73 reference genome (Zm-B73-REFERENCE-NAM-5.0) using HISAT2 with default parameters. Gene expression levels were quantified as fragments per kilobase of transcript per million mapped reads (FPKM). Differentially expressed genes (DEGs) were identified using DESeq2, with an adjusted *p* value (false discovery rate, FDR) < 0.05 and |log2(fold change)| ≥ 1.0 considered statistically significant. Transcriptome data were subsequently analyzed to characterize the expression profiles of ZmTALE genes under low-phosphorus (LP) stress. Gene expression patterns were visualized as heatmaps using the Heatmap Illustrator module implemented in TBtools II, enabling comparative analysis of the transcriptional responses of ZmTALE genes under LP conditions.

To validate the RNA-seq results, the expression levels of selected ZmTALE genes were further examined by real-time quantitative PCR (RT-qPCR). Total RNA was reverse-transcribed into first-strand cDNA, and *ZmActin3* was used as the internal reference gene. The primer sequences used for RT-qPCR are listed in Supplementary Table S3 Each experiment included three independent biological replicates. Quantitative PCR was performed in a 20 μL reaction volume under the following cycling conditions: 95 °C for 30 s, followed by 40 cycles of 95 °C for 30 s, 60 °C for 20 s, and 72 °C for 30 s. A melting curve analysis was subsequently conducted by increasing the temperature from 60 °C to 95 °C at 0.5 °C increments to verify amplification specificity. Relative gene expression levels were calculated using the 2^^*−ΔΔCt*^ method [47].

### 4.8. Determination of Shoot Dry Weight, Acid Phosphatase Activity, and Inorganic Phosphate Content

The long-term LP treatment materials were also used for measurement of phosphorus-related physiological indicators. After 14 days of LP treatment, whole seedlings were harvested for the determination of shoot length, shoot dry weight, acid phosphatase (APA) activity, and inorganic phosphate (Pi) content.

For shoot dry weight measurement, shoots were dried in a forced-air oven at 65 °C until a constant weight was reached, and the dry weight was recorded using an analytical balance.

APA activity was determined according to the method of Tabatabai and Bremner (1969) [48] with minor modifications. Briefly, 1.0 g of fresh leaf tissue was homogenized in 2 mL of 0.2 M sodium acetate buffer and centrifuged at 13,400× *g* for 20 min at 4 °C. The resulting supernatant was collected for enzyme activity determination following the protocol described by Yun and Kaeppler (2001) [49]. APA activity was expressed on a fresh weight basis.

The Pi concentration was determined as previously described by Nanamori et al. (2004) [50]. Approximately 0.05 g of fresh leaf tissue was incubated in 2 mL of 2% (*v*/*v*) acetic acid at 42 °C for 30 min, followed by centrifugation at 13,400× *g* for 2 min. The supernatant was collected, and Pi concentration was quantified using the molybdenum blue colorimetric method as described by Katewa and Katyare (2003) [51]. Absorbance was measured at 820 nm using a spectrophotometer (TFN, Shanghai, China), and Pi concentration was calculated based on a standard curve generated with serial concentrations of KH_2_PO_4_.

All measurements were performed using three independent biological replicates, with each replicate consisting of pooled tissues collected from at least three individual seedlings.

### 4.9. Subcellular Localization of ZmTALE Proteins

The full-length coding sequences (CDSs) of ZmTALE10, ZmTALE14, and ZmTALE31, excluding the stop codon, were amplified from maize cDNA using gene-specific primers (Supplementary Table S3) and cloned into the pCAMBIA1300-GFP vector containing the green fluorescent protein (GFP) reporter gene under the control of the Cauliflower mosaic virus (CaMV) 35S promoter, generating the 35S::ZmTALE-GFP fusion constructs.

The recombinant plasmids, together with the empty 35S::GFP vector as a control, were individually introduced into Agrobacterium tumefaciens strain GV3101. Agrobacterium-mediated transient transformation of Nicotiana benthamiana leaf epidermal cells was performed as previously described [52]. After agroinfiltration, plants were maintained in darkness for 24 h and subsequently grown under normal growth conditions for 72 h. GFP fluorescence signals were observed using a Leica laser scanning confocal microscope (Leica Microsystems, Wetzlar, Germany) with excitation at 488 nm.

### 4.10. Yeast Transcriptional Activation Assay

To evaluate the transcriptional activation activities of ZmTALE10, ZmTALE14, and ZmTALE31, their full-length CDSs were individually cloned into the pGBKT7 vector to generate fusion constructs with the GAL4 DNA-binding domain (BD).

The recombinant plasmids and the empty pGBKT7 vector (negative control) were transformed into the Saccharomyces cerevisiae strain Y2HGold using the polyethylene glycol/lithium acetate (PEG/LiAc) transformation method [53]. Positive transformants were first selected on synthetic dropout (SD) medium lacking tryptophan (SD/-Trp). To assess transcriptional activation activity, successfully transformed yeast colonies were subsequently transferred onto SD medium lacking tryptophan, histidine, and adenine (SD/-Trp/-His/-Ade) supplemented with X-α-Gal. Colony growth and blue color development were examined after incubation at 30 °C for 3–5 days. Growth accompanied by blue coloration indicated that the corresponding ZmTALE protein possessed intrinsic transcriptional activation activity.

### 4.11. Statistical Analysis

All experiments were performed with three independent biological replicates, and the data are presented as the mean ± standard deviation (SD). Statistical analyses were conducted using SPSS Statistics 27.0. Differences among groups were evaluated by two-way analysis of variance (ANOVA). When a significant treatment effect was detected (*p* < 0.05), multiple comparisons were performed using the least significant difference (LSD) test, and the Waller-Duncan multiple range test was additionally applied to verify the robustness of the statistical results. Differences were considered statistically significant at *p* < 0.05. In all bar charts, different lowercase letters indicate statistically significant differences among groups (*p* < 0.05), whereas identical lowercase letters indicate no significant differences (*p* ≥ 0.05).

## 5. Conclusions

In this study, we identified 40 TALE family members in the maize genome and performed a comprehensive genome-wide characterization, including analyses of their phylogenetic relationships, chromosomal distribution, gene structures, conserved motifs, gene duplication events, syntenic relationships, and promoter cis-regulatory elements. Promoter analysis revealed numerous cis-acting elements associated with phytohormone responsiveness and abiotic stress, suggesting that ZmTALE genes may participate in diverse biological processes. Expression profiling based on RNA-seq and qRT-PCR identified several *ZmTALE* genes that were differentially expressed under low-phosphorus conditions, with *ZmTALE10*, *ZmTALE14*, and *ZmTALE31* exhibiting distinct transcriptional responses. In addition, subcellular localization assays demonstrated that these three proteins are localized in the nucleus, and yeast transcriptional activation assays confirmed that they possess transcriptional activation activity, providing experimental evidence that they function as nuclear transcription factors.

Overall, this study provides a comprehensive overview of the evolutionary characteristics and expression profiles of the maize TALE gene family and identifies LP-responsive TALE genes as promising candidates for future functional studies to elucidate their molecular roles in maize responses to phosphorus deficiency.

## Figures and Tables

**Table 1:** The detailed information of TALE members.

Gene	Gene ID	CDS (bp)	Amino Acid (aa)	PI	MW	Instability Index	Aliphatic Index	GRAVY	Subcellular Localization
ZmTALE1	Zm00001eb001720	933	310	5.73	34,336.78	59.64	75.61	−0.56	nucl.
ZmTALE2	Zm00001eb001770	1863	620	6.16	66,061.11	49.11	68.18	−0.49	nucl.
ZmTALE3	Zm00001eb005020	1764	587	5.82	62,181.77	49.04	67.31	−0.44	nucl.
ZmTALE4	Zm00001eb013560	1797	598	6.69	64,715.13	57.27	88.63	−0.21	E.R.
ZmTALE5	Zm00001eb021970	1839	612	6.34	64,715.25	47.69	69.69	−0.34	nucl.
ZmTALE6	Zm00001eb031950	285	95	4.88	10,998.39	41.24	78.11	−0.49	cyto
ZmTALE7	Zm00001eb032780	2004	668	5.76	72,679.92	57.87	64.64	−0.63	nucl.
ZmTALE8	Zm00001eb051900	1956	651	5.68	71,815.74	49.81	63.16	−0.69	nucl.
ZmTALE9	Zm00001eb051910	1125	374	9.07	40,996.23	60.77	84.47	−0.52	cyto
ZmTALE10	Zm00001eb052120	999	332	5.96	36,589.86	49.75	65.66	−0.73	nucl.
ZmTALE11	Zm00001eb055920	1080	359	6.41	39,826.68	52.36	73.43	−0.64	nucl.
ZmTALE12	Zm00001eb055940	1083	360	6.03	39,643.75	39.64	69.00	−0.52	nucl.
ZmTALE13	Zm00001eb056260	2268	755	6.49	79,167.79	51.69	62.07	−0.50	nucl.
ZmTALE14	Zm00001eb058930	1095	364	5.91	40,276.27	56.65	62.88	−0.64	nucl.
ZmTALE15	Zm00001eb108700	351	116	4.97	13,112.02	39.85	91.72	−0.23	cyto, chlo
ZmTALE16	Zm00001eb116630	1089	362	8.32	40,246.68	38.40	79.50	−0.55	chlo
ZmTALE17	Zm00001eb117820	1083	360	6.23	39,215.02	40.51	64.72	−0.63	nucl.
ZmTALE18	Zm00001eb130180	888	295	5.51	32,493.47	42.82	68.51	−0.55	nucl.
ZmTALE19	Zm00001eb147970	1764	587	7.29	63,158.83	54.00	68.07	−0.44	nucl.
ZmTALE20	Zm00001eb181490	411	136	4.91	15,591.79	43.13	96.76	−0.21	cyto
ZmTALE21	Zm00001eb202140	2016	671	6.06	69,914.65	43.61	62.43	−0.41	nucl.
ZmTALE22	Zm00001eb206760	945	314	6.02	34,111.33	45.09	71.34	−0.51	nucl.
ZmTALE23	Zm00001eb217350	1920	639	5.46	70,848.35	49.61	63.87	−0.71	nucl.
ZmTALE24	Zm00001eb217470	492	163	4.59	18,005.31	54.09	77.98	−0.33	mito, chlo
ZmTALE25	Zm00001eb217480	645	214	7.77	24,066.87	48.30	67.57	−0.76	nucl.
ZmTALE26	Zm00001eb234270	903	284	5.89	32,669.76	45.98	74.72	−0.44	nucl.
ZmTALE27	Zm00001eb239680	1620	539	6.73	56,452.11	61.11	68.78	−0.25	nucl.
ZmTALE28	Zm00001eb264910	897	298	5.32	32,662.69	44.76	65.91	−0.58	nucl.
ZmTALE29	Zm00001eb268690	1497	514	6.68	54,229.07	50.83	71.48	−0.44	nucl.
ZmTALE30	Zm00001eb289480	1731	576	8.29	61,910.33	55.50	68.87	−0.45	nucl.
ZmTALE31	Zm00001eb299420	1056	351	6.4	38,800.64	45.60	64.70	−0.68	nucl.
ZmTALE32	Zm00001eb322410	453	150	6.29	16,469.00	45.86	104.07	−0.04	cyto
ZmTALE33	Zm00001eb347710	1779	592	9.24	64,138.12	51.74	70.12	−0.45	nucl.
ZmTALE34	Zm00001eb354670	504	167	5.48	18,305.62	49.61	81.80	−0.42	nucl, chlo
ZmTALE35	Zm00001eb354880	984	327	5.27	35,593.02	54.47	67.25	−0.51	nucl.
ZmTALE36	Zm00001eb384240	1476	491	7.19	52,469.65	56.18	66.86	−0.32	nucl.
ZmTALE37	Zm00001eb386830	951	316	5.69	34,522.64	46.69	69.05	−0.56	nucl.
ZmTALE38	Zm00001eb403720	957	309	5.64	34,768.18	60.95	81.20	−0.41	nucl.
ZmTALE39	Zm00001eb437470	282	93	9.23	10,577.17	51.38	82.90	−0.61	cyto, mito
ZmTALE40	Zm00001eb438600	282	93	9.23	10,547.08	49.77	82.90	−0.63	cyto, mito

## Data Availability

The raw data of low-phosphorus treatment have been deposited in the Genome Sequence Archive of the BIG Submission Portal, National Genomics Data Center (NGDC, https://ngdc.cncb.ac.cn/) under the Bioproject (PRJCA044786).

## Supplementary Materials

The following supporting information can be downloaded at: https://www.mdpi.com/article/10.3390/plants15162444/s1. Figure S1. Predicted 3D structures of other maize TALE proteins. Blue color represents the N-terminus of the amino acid sequence, and red color represents the C-terminus of the amino acid sequence. Then, the blue part is an alpha-helix. Figure S2. Phylogenetic analysis of the TALE gene family in seven species. An phylogenetic tree was constructed using the neighbor-joining (NJ) method with 1000 bootstrap replicates. The outer ring colors represent four subfamilies: BEL1-like (pink), KNOX I (cyan), KNOX II (purple), KNOX III (yellow). Different shapes and colors represent different species: a red pentagram represents maize (*Zea mays*), a green dot represents Arabidopsis thaliana, a blue triangle represents sorghum (*Sorghum bicolor*), a yellow square represents rice (*Oryza sativa*), a pink triangle represents sugarcane (*Saccharum officinarum*), a blue dot represents poplar (*Populus trichocarpa*), and a purple triangle represents grape (*Vitis vinifera*). Table S1. Prediction of Secondary Structure of maize TALE Proteins. Table S2. Ka/Ks analysis of the syntenic ZmTALE gene pairs. Table S3. The primers used in this study.
