# Supplementary material for: Genome-Wide Identification of the Maize TALE Gene Family and Their Expression Analysis Under Low-Phosphorus Response in Maize (Zea mays L.)
Source: Plants (Basel). 2026 Aug 11;15(16):2444. doi: 10.3390/plants15162444 (PMC13516468; doi:10.3390/plants15162444)
Supplement: Supplementary file 1 [file plants-15-02444-s001.zip › Supplementary Figures.pdf]

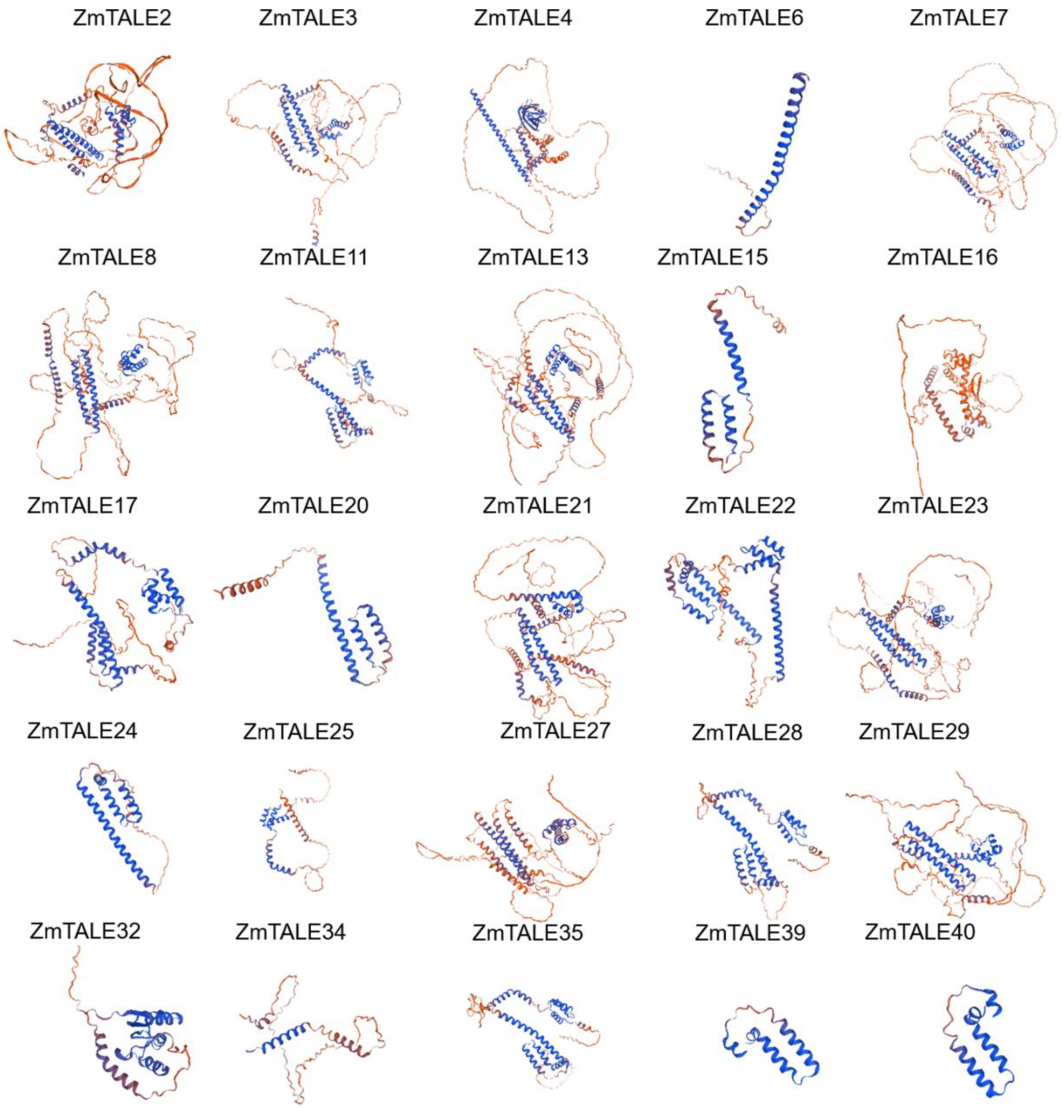

Figure S1. Predicted 3D structures of other maize TALE proteins. Blue color represents the N-terminus of the amino acid sequence, and red color represents the C-terminus of the amino acid sequence. Then, the blue part is an alpha-helix.

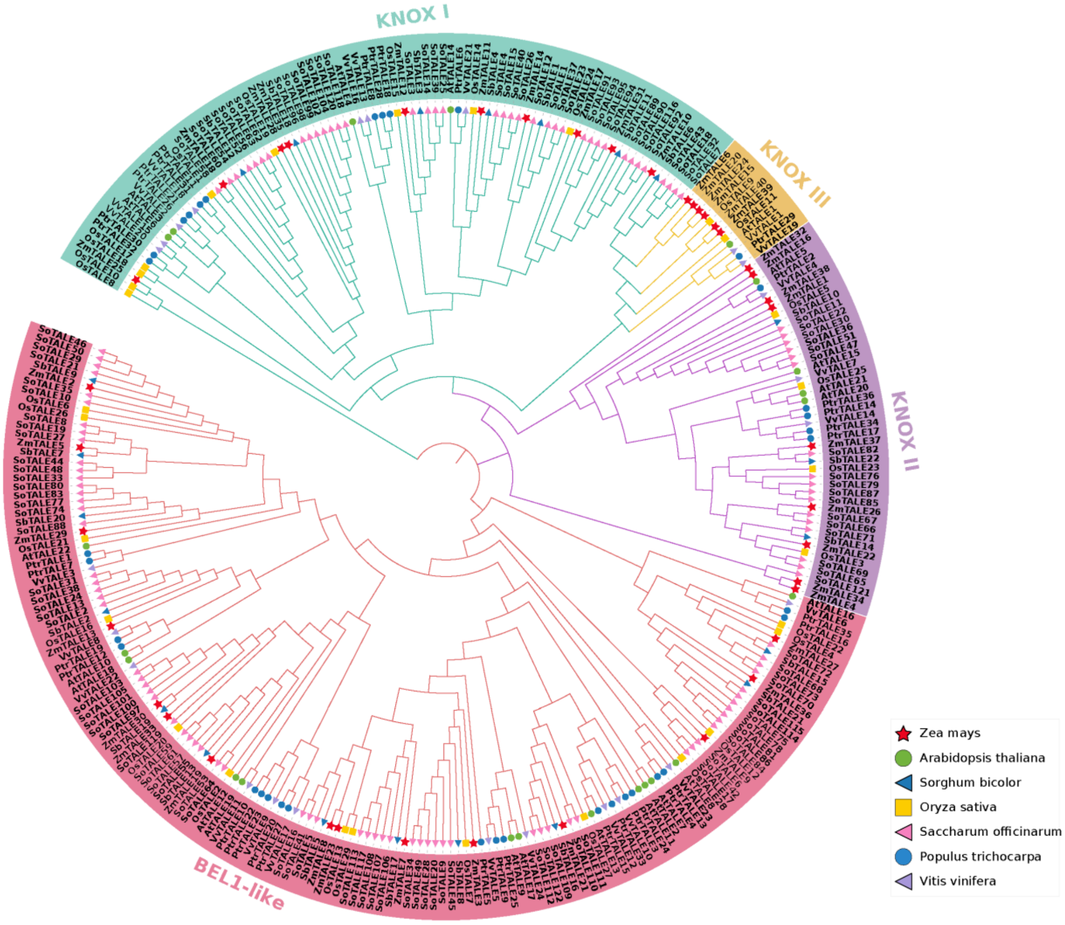

Figure S2. Phylogenetic analysis of the TALE gene family in seven species. An phylogenetic tree was constructed using the neighbor-joining (NJ) method with 1000 bootstrap replicates. The outer ring colors represent four subfamilies: BEL1-like (pink), KNOX I (cyan), KNOX II (purple), KNOX III (yellow). Different shapes and colors represent different species: a red pentagram represents maize (Zea mays), a green dot represents Arabidopsis thaliana, a blue triangle represents sorghum (Sorghum bicolor), a yellow square represents rice (Oryza sativa), a pink triangle represents sugarcane (Saccharum officinarum), a blue dot represents poplar (Populus trichocarpa), and a purple triangle represents grape (Vitis vinifera).
